# Evolution and Reconstruction of Air‐Electrode Surface Composition in Reversible Protonic Ceramic Cells: Mechanisms, Impacts on Catalytic Performance, and Optimization Strategies – A Review

**DOI:** 10.1002/adma.202416528

**Published:** 2025-02-05

**Authors:** Nai Shi, Yun Xie, Moses Oludayo Tadé, Zongping Shao

**Affiliations:** ^1^ WA School of Mines: Minerals, Energy and Chemical Engineering (WASM‐MECE) Curtin University Perth WA 6102 Australia; ^2^ Department of Energy Conversion and Storage Technical University of Denmark Kgs Lyngby 2800 Denmark

**Keywords:** advanced characterization, air‐electrode, performance degradation, proton ceramic cells, surface evolution

## Abstract

Reversible protonic ceramic cells (R‐PCCs) are at the forefront of electrochemical conversion devices, capable of reversibly and efficiently converting chemical energy into electricity at intermediate temperatures (350–700 °C) with zero carbon emissions. However, slow surface catalytic reactions at the air‐electrode often hinder their performance and durability. The electrode surface is not merely an extension of the bulk structure, equilibrium reconstruction can lead to significantly different crystal‐plane terminations and morphologies, which are influenced by material's intrinsic properties and external reaction conditions. Understanding electrode surface evolution at elevated temperatures in water‐containing, oxidative atmospheres presents significant importance. In this review, a comprehensive summary of recent processes in applying advanced characterization techniques for high‐temperature electrode surfaces is provided, exploring the correlations between surface evolution and performance fluctuations by examining the structural evolution and reconstruction of various air‐electrode surfaces associated with degradation and activation phenomena, offering insights into their impact on electrode performance. Furthermore, reported strategies and recent advances in enhancing the electrochemical performance of R‐PCCs through engineering air‐electrode surfaces is discussed. This review offers valuable insights into surface evolution in R‐PCCs and is expected to guide future developments in high‐temperature catalysis, solid‐state ionics, and energy materials.

## Graphical abstract

This review comprehensively summarizes the surface evolution and reconstruction of reversible protonic ceramic cell (R‐PCC) air‐electrodes. It begins by analyzing the thermodynamic and kinetic contributions to surface evolutions, followed by a summary of factors that lead to electrochemical performance degradation, emphasizing on both the negative and positive effects of water. Additionally, recent advances in enhancing electrode surface activity are presented.

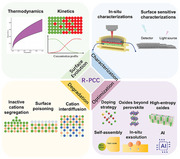

## 1 Introduction

As the importance of renewable and sustainable energies such as solar, wind, and hydroelectric powers continue to increase for realizing a carbon‐neutral society, the prospects for building a renewable and sustainable energy system are highly promising. However, these intermittent natural resources suffer from regional, seasonal, or time limitations, resulting in fluctuating electricity outputs.^[^ ^1^ ^]^ The development of efficient and reliable energy conversion devices that can reversibly convert chemical energy into electricity becomes crucial.^[^ ^2^ ^]^ Reversible protonic ceramic cells (R‐PCCs), featuring an all‐solid‐state structure with proton‐conducting oxides as electrolytes, represent the latest technology for converting chemical energy into electricity with potentially high efficiency and low costs.^[^ ^3^ ^]^ Over other energy conversion technologies, R‐PCCs offer distinct advantages, including the no requirement of precious metals, quiet operation, and distributed power generation.^[^ ^4^ ^]^ The R‐PCC electrochemical performance, unfortunately, usually limited by its sluggish proton‐related air‐electrode reactions, which integrates oxygen reduction reaction (ORR, Equation 1) in protonic ceramic fuel cell (PCFC) mode, and oxygen evolution reaction (OER, Equation 2) in protonic ceramic electrolysis cell (PCEC) mode.

(1) $$4{\mathrm{OH}}_{O}^{\cdot }+O_{2}+4e^{\prime }\to 2H_{2}O+4O_{O}^{X}$$

(2) $$2H_{2}O+4O_{O}^{X}\to 4{\mathrm{OH}}_{O}^{\cdot }+O_{2}+4e^{\prime }$$

Surface reaction processes, such as reactants adsorption, charge transfer, dissociation, and desorption of products, govern the air‐electrode reaction kinetics and ultimately the overall out‐put performance of R‐PCC,^[^ ^3^, ^5^ ^]^ they are closely linked to the properties of the air‐electrode surface. However, unlike a perfect crystal cleave, the catalyst surface atoms exhibit unsaturated bonds, leading to higher free energy compared to the ideal bulk lattice. To minimize this energy, the lattice tends to expose terminated surfaces, which promote cation segregation and undergo in situ exsolution of nanoparticles. Analyzing such surfaces evolution processes and developing efficient, stable electrodes, however, face several key challenges, such as unclear surface evolution and reconstruction mechanisms and complex electrochemical performance degradation behavior. The surface evolution is influenced by a combination of intrinsic materials properties (i.e., cation characteristics, lattice strain, surface electrostatics) and external conditions (i.e., temperature, oxygen partial pressure, steam atmosphere). Isolating specific factors from the complicated reactions is challenging. Thus, understanding surface evolution processes requires a deep knowledge of in situ and ex situ characterization techniques, as well as electrochemical characterization models. High operation temperatures, harsh reaction environments, and the dynamic nature of material surfaces all contribute to the degradation of electrochemical performance. Air‐electrode surface deterioration is primarily caused by inactive cation surface segregation, poisoning by strongly adsorbed species, and cation interdiffusion between neighboring components. In particular, the presence of water further complicates surface evolution, making it crucial to understand the effects of water on oxygen‐related reactions (ORR/OER) and surface degradation.^[^ ^4^, ^6^ ^]^

Over the past few decades, various strategies have been employed to improve surface reaction kinetics in R‐PCCs. These include doping strategies,^[^ ^7^ ^]^ the development of oxides beyond perovskites,^[^ ^8^ ^]^ surface engineering through the construction of core‐shell nano catalysts,^[^ ^9^ ^]^ self‐assembly,^[^ ^10^ ^]^ in situ exsolution of nanoparticles,^[^ ^11^ ^]^ and the use of artificial intelligence (AI) to identify and predict stable and efficient surfaces.^[^ ^12^ ^]^ The principle ideas behind these approaches are to tailoring surface compositions, furnishing more reaction sites,^[^ ^11^, ^13^ ^]^ and altering surface electronic structures.^[^ ^14^ ^]^ For instance, constructing electrodes to form active nanophases through in situ exsolution or self‐assembly has been proved to effectively enhance electrochemical performance.^[^ ^15^ ^]^ Tailoring surface acidity has also been found to improve surface activity. For example, it was found that infiltrating basic oxides of Li_2_O, CaO, Gd_2_O_3_ onto Pr_0.1_Ce_0.9_O_2‐δ_ enhanced the surface reactivity. This improvement was attributed to the lower work functions of the basic oxides than Pr_0.1_Ce_0.9_O_2‐δ_, which cause downward band bending and the accumulation of electrons at the surface, thus facilitating charge transfer processes and improved the surface activity of the electrodes.^[^ ^16^ ^]^ Recent reports show that accelerating air‐electrode surface step limitation reactions and engineering the electrolyte/electrode interface can boost electrochemical performance as low as 350 °C.^[^ ^3^, ^17^ ^]^

However, conflicting results and scattered data are frequently observed in previously reported findings. For instance, some studies indicate that the presence of water exacerbates cathode poisoning, leading to faster performance decay.^[^ ^18^ ^]^ Conversely, other reports suggest that water enhances surface catalytic reactions and promotes active phase formation.^[^ ^6^, ^15^, ^19^ ^]^ It is generally accepted that air‐electrodes degrade with high‐temperature annealing due to accelerated cation diffusion and inactive phase formation.^[^ ^20^ ^]^ However, some studies show that high‐temperature treatment boost electrochemical performance through inducing the active nanophases formation.^[^ ^10^, ^21^ ^]^ With same air‐electrode, electrochemical performance data collected by different research groups and at different times often show variability. This inconsistency largely arises from the complex and dynamic nature of the electrode surface, highlighting the need for systematic analysis and summarization to better understand and predict air‐electrode behavior.

**Figure 1:**
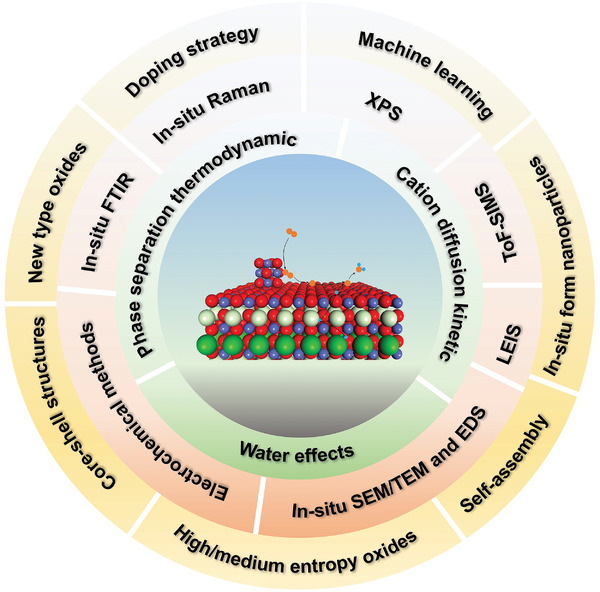
Schematic illustration of major contents in this review.

This article provides an in‐time comprehensive review in the recent advances in understanding of the evolution and reconstruction of air‐electrode surface composition in R‐PCCs, its impact on catalytic performance, and the available optimization strategies (**Figure** **1**). We delve into the intricacies of air‐electrode surface evolution and reconstruction mechanisms, exploring them from both macro and micro perspectives. Special attention is paid to water influence on air‐electrode reactions and the surface degradation mechanisms, including inactive cation surface segregation, surface poisoning, cation interdiffusions, and its contributions to surface reconstructions. We then provide a summary of the wide spectrum of techniques for analyzing material surfaces, including in situ characterization techniques and surface‐sensitive ex situ characterizations. The latest surface modification methods, with emphasis on developing new oxides, in situ reconstruction, self‐assembly techniques, and machine‐learning based techniques, as potential solutions to enhance the performance and longevity of air‐electrodes, are provided. Finally, some perspectives for further in‐depth understanding of the evolution and reconstruction processes of air‐electrode in R‐PCCs and new performance optimization strategies are proposed.

## 2 Surface Evolution on R‐PCC Air‐Electrode

The idealized surface is flat, rigid, perfectly smooth, assumed to have homogeneously distributed atoms. It is represented as a semi‐infinite crystal, where the atom positions and the periodicity of the structure are identical to those of the original crystal. However, this idealized surface primarily exists in modeling simulations and is rarely observed in real‐world conditions, it overlooks several critical factors, including the interruption of the periodic potential at the crystal surface, the thermal motion of surface atoms, thermal diffusion, defects, and the influence of the external environment on the surface. In reality, the surface of practical electrodes is often non‐ideal and can undergo surface evolution as a result of the material's intrinsic properties or external conditions. Thermodynamic and/or kinetic factors govern this process, enabling electrode materials' prediction, design, and optimization through systematic simplification and analysis of the relevant parameters across different (materials) systems. Here, we briefly discuss the thermodynamic factors that affect crystal plane orientation, phase separation, and kinetics for cation diffusions.

### 2.1 Surface Evolution Thermodynamics

Thermodynamics determines the final stable state of electrode surface evolution under fixed conditions, but it may take an extremely long time when the temperature is low. In real crystalline lattices, the presence of unsaturated bonds on the surface atoms makes them stay at a high free energy state. To minimize this free energy, the surface tends to interact with other lattice defects, such as dislocations, vacancies, and foreign atoms.^[^ ^22^ ^]^ The phase stability is associated with chemical potential of subcomponents of the material, and external temperatures and pressures. A multi‐component system can be evaluated using the equation ∆G_mix_ = ∆H_mix_ – T∆S_mix_, where ∆G_mix_, ∆H_mix_, and ∆S_mix_ are Gibbs free energy, mixing enthalpy, and mixing entropy, respectively. When the bulk Gibbs free energy is less than zero, it indicates that the phase is thermodynamically stable and the components are well‐mixed without formation of secondary phases. Inactive cation segregations are commonly encountered in many oxides when annealed at elevated temperatures.^[^ ^23^ ^]^ For example, annealing La_0.6_Sr_0.4_CoO_3‐δ_ (LSC) at 650 °C results in the structure transformation from Sr‐enriched surface to a SrO/Sr(OH)_2_‐rich, phase‐seperated state, which negatively impacts catalytic performance.^[^ ^23a^ ^]^ The thermodynamic parameters for this segregation can be obtained experimentally. Fister et al. perfromened Sr segregation investigations on (001) oriented La_0.7_Sr_0.3_MnO_3_ thin film using synchrotron‐based total reflection x‐ray fluorescence (TXRF) measurments in a wide temperature range of 25–900 °C and oxygen partial pressures (*p*O_2_) range of 0.15–150 Torr, the Arrhenius plots between surface Sr segreagtion against *p*O_2_ extracts enthalpy of segreagtion Δ*H*_seg_. They found the exothermic heat of segreagtion changes from −9.5 to −2.0 kJ mol^−1^ with increasing *p*O_2_. The enhanced Sr surface segreagtion under lower *p*O_2_ may result from the electrostatic interactions between positive charged oxygen vacancies and negative charged $Sr_{La}^{,}$.^[^ ^24^ ^]^

**Figure 2:**
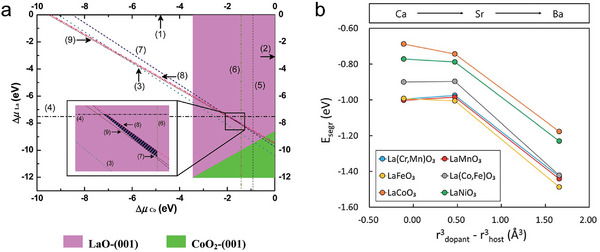
a) Phase diagram for the outermost‐layer Sr‐doped (001) surfaces with different terminations of cubic La_0.875_Sr_0.125_CoO_3_ system at 1100 K and oxygen partial pressure of 0.2 atm. The numbers in parentheses point to lines, where phase separations of metals and their oxides from bulk La_0.875_Sr_0.125_CoO_3_ begin to occur: 1) La, 2) Co, 3) Sr, 4) La_2_O_3_, 5) CoO, 6) Co_3_O_4_, 7) SrO, 8) LaCoO_3_, 9) SrCoO_3_. Reproduced with permission.^[^ ^25^ ^]^ Copyright 2015, Royal Society of Chemistry. b) Surface segregation energy against size mismatch between the A‐site dopant and host cations. Reproduced with permission.^[^ ^28^ ^]^ Copyright 2016, Royal Society of Chemistry.

The advancing of computional calculations offers feasible way for predicting the stable exposed crystal planes, which generally follows two principles: 1) It is in equilibrium with both the surrounding oxygen atmosphere and the bulk material, 2) it has the lowest surface Gibbs free energy.^[^ ^25^ ^]^ In case of air‐electrodes in PCCs, stability in steam‐containing atmospheres is also a critical requirement. Park et al. calculated the surface Gibbs free energy on La_0.5_Sr_0.5_FeO_3_ (LSF) material at elevated temperatures based on density functional theory (DFT) using the equation of Ω^*i*^ = $\frac{1}{2A_{s}}\;\left[G_{slab}^{i}-N_{La}^{i}\mu_{La}-N_{Sr}^{i}\mu_{Sr}-N_{Fe}^{i}\mu_{Fe}-N_{O}^{i}\mu_{O}\right]$, where the $G_{slab}^{i}$ is the Gibbs energy of i‐terminated surface, A_s_ is the area of the slab, N is the number of atoms in the slab and µ is the the chemical potential of each species in the bulk LSF. They show as the temperatue increases, the SrO terminated surface becomes more stable than FeO_2_‐La terminated surface, predicting formation of SrO terminated surface becomes dominate at high temperatures and is in agreement with experiment results.^[^ ^26^ ^]^ Similiarly, through compartive calculations between cubic LaCoO_3_ and La_1‐x_Sr_x_CoO_3_ surfaces, it was found both CoO_2_‐termined and LaO‐termined (001) surfaces can be stable from 750 to 1250 K on LaCoO_3_. In terms of La_0.875_Sr_0.125_CoO_3_, it is stable only within a small area with narrow Δµ_*Co*_ and Δµ_*La*_ in phase diagram, and under real R‐PCC operations, it falls into the La_0.75_Sr_0.25_O‐terminated (001) surface, proving the Sr‐doping in LaCoO_3_ destablized the CoO_2_‐terminated surface (**Figure** **2**a).^[^ ^25^, ^27^ ^]^

The thermodynamic calculations also used for predicting phase seperations. The intermediate cation segregation energy is determined using the equation E_segr._ = E_surf._‐E_bulk_, where E_segr._ represents the surface segregation energy, E_bulk_, and E_surf._ are the total energies of the slab when the host cations replace the dopant cations at the bottom and top of the A‐O layer, respectively. The DFT calculations investigated on several A‐site doped oxides, including LaACr_0.5_Mn_0.5_O_3_, LaAMnO_3_, LaAFeO_3_, LaCo_0.25_Fe_0.75_O_3_, LaACoO_3_, and LaANiO_3_, where A represents Ca, Sr, or Ba. It was found the elastic energy caused by the size mismatch between A and B site cations is the dominate factor that governs segregation energy. The bigger difference between dopants and host cations, as well as smaller free volumes, resulted in the higher elastic interactions of dopant cations and a higher tendency of segregation (Figure 2b).^[^ ^28^ ^]^ However, these calculations are typically conducted using 0K DFT formation energy and are highly sensitive to parameters such as the vibrational frequency of solids, temperature, zero‐point energy corrections, and phonon calculations. Comprehensive consideration to calculation details, along with strategically experiments, is critical for understanding the stable phases under specific conditions.

The air‐electrode in R‐PCCs could face more complicated situations becasue it naturally working in humidified conditions, and such moistured atmospheres may accelerate the cation segreagtion.^[^ ^29^ ^]^ The Near‐ambient pressure XPS (NAP‐XPS) experiments on La_0.5_Sr_0.5_FeO_3‐δ_ thin film verified Sr segration to the surface in a humidfied condition, accompanied by the increasing in the polarization resistance.^[^ ^30^ ^]^ PrBa_0.5_Sr_0.5_Co_1.5_Fe_0.5_O_5+δ_ (PBSCF) has been demonstrated as a good air‐electrode for R‐PCCs for its proton uptake and conduction ability, as well as great catalytic performance toward ORR and OER.^[^ ^3d^ ^]^ However, electrochemcial characterizations together with scanning electron microscope tests on PBSCF thin film showed wet PBSCF exhibited ≈4 times more rapid degradation in the area specific resistance values with ≈4 times higher coverage of Ba and Sr enriched clusters at the surface. One possible reason behind this is increased oxygen vacancies in humidified atmospheres, which in turn create higher electrostatic attraction to the doped cations toward the surface.^[^ ^29a^ ^]^ Sharma et al. combined thermodynamic calculations of cation segregation with experimental verifications on (La, A)MnO_3_ (A = Ca, Sr, and Ba) (001) surfaces. Their results demonstrate all dopants promotes the segreagtion process across a wide range of temperatures and water partial pressures, and atomic force microscopy (AFM) analysis confirmed humidified atmosphere promotes cation segreagtion. It was proposed that interplay of oxygen vacancies and water molecules further drives surface segregation.^[^ ^31^ ^]^

### 2.2 Cation Diffusion Kinetics

**Figure 3:**
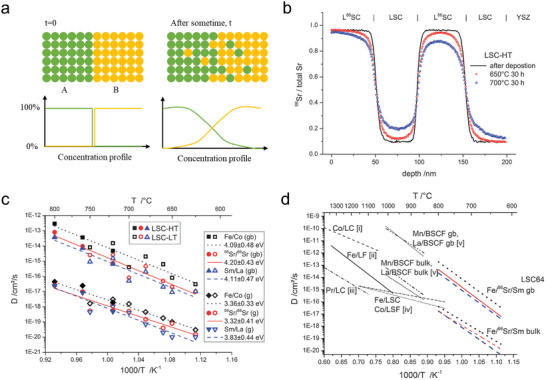
a) Schematic diagram of an inter‐diffusion process, indicated by the gradient in concentration of species A and B, redraw from literature with permission.^[^ ^37^ ^]^ Copyright 2023, Elsevier. b) The normalized values c*_Sr_* in diffusion profiles of multilayered L^86^SC|LSC. c) Arrhenius plot of tracer diffusion coefficients at grains (g) and grain boundaries (g_b_). d) Comparison of reported diffusion coefficients values for mixed conducting perovskite‐type oxides, the data obtained by different methods; solid state reaction: i) Co in LaCoO_3_,^[^ ^32b^ ^]^ ii) Fe in LaFeO_3_;^[^ ^32c^ ^]^ impurity diffusion: iii) Pr in LaCoO_3_,^[^ ^36^ ^]^ iv) Co in La_0.8_Sr_0.2_FeO_3‐δ_ and Fe in La_0.8_Sr_0.2_CoO_3‐δ_,^[^ ^38^ ^]^ v) La and Mn in Ba_0.5_Sr_0.5_Co_0.8_Fe_0.2_O_3‐δ_.^[^ ^33^, ^34^ ^]^ Reproduced with permission.^[^ ^34^ ^]^ Copyright 2013, Royal Society of Chemistry.

At high temperatures, cation diffusion kinetics play a dominant role in determining phase composition, becasue thermodynamic equilibrium is achieved more rapidly under such condition. Diffusion coefficients and activation energy are two important parameters for determing cation diffusions. Two experimental approaches are commonly used to investigate cation diffusion coefficients in perovskite oxides. The first method involves using diffusion couples with two polished bulk samples connect together and annealed at high temperatures (**Figure** **3**a).^[^ ^32^ ^]^ The second approach uses a single polished sample with a thin tracer layer applied on its surface.^[^ ^33^ ^]^ The sample is annealed for a period at a constant temperature, after which the resulting cation distribution is analyzed using characterizaion techniques such as secondary ion mass spectroscopy, energy dispersive X‐ray spectroscopy, or by detecting radiation from radioactive tracer isotopes.^[^ ^34^ ^]^ Furthermore, in order to seperate the grain interior and grain boundary diffusion coefficients, numberical models are included when investigating diffusion properties in polycrystal materials.

An example is the investigation of cation diffusion in LSC by fabricating LSC thin films on a yttria‐stabilized zirconia (YSZ) (100) surface and using isotopic ^86^Sr along with tracer elements such as Fe and Sm to establish cation diffusion profiles. The samples were annealed at elevated temperatures (Figure 3b). Fick's second law, ($\frac{\partial c}{\partial t}$) = ∇(*D*∇c) was employed to determine the diffusion coefficient D*, and a brick‐layer model was used to distinguish grain and grain boundary diffusion coefficients. The study found diffusion energy barriers of ≈4.1 eV for grain boundaries and 3.5 eV for grains, with cation diffusion in the grains being about three orders of magnitude slower than in the grain boundaries (Figure 3c). The authors suggested that differences in formation enthalpy for cation defects between grains and grain boundaries could explain this discrepancy. Another key finding was that although B‐site Co/Fe exhibited higher diffusion coefficients, the diffusion coefficients for all cations were found to be within one order of magnitude and shared similar activation energies (Figure 3c,d). The results indicate that a defect complex, comprising both an A‐site and a B‐site vacancy, governs the mobility of both A‐site and B‐site cations and lead to similar activation energies and diffusion coefficients for all cations.^[^ ^34^ ^]^

The material point defects and lattice structure have dominate influence to the cation diffusion kinetics. Palcut et al. investigated the diffusion of Pr^3+^ in LaCoO_3_, LaMnO_3_, and LaFeO_3_ materials by applying a praseodymium oxide onto the surface of LaMnO_3_, LaCoO_3_, and LaFeO_3_, followed by annealing at 1373, 1473, 1573, and 1673 K for 20, 5, 2, and 1 h(s) respectively. The cation distributions were analyzed using SIMS characterization. They found that Pr^3+^ diffuses faster in LaMnO_3_ than in LaCoO_3_ and LaFeO_3_, which was attributed to the higher intrinsic point defects in LaMnO_3_ compared to the other two investigated materials, and cubic structured LaMnO_3_ facilitates Pr^3^⁺ cation diffusion more effectively than the orthorhombic LaFeO_3_ and rhombohedral LaCoO_3_ oxides. Further, calculated using the Whipple‐Le Claire equation^[^ ^35^ ^]^ by assuming a grain boundary thickness of 1 nm, the diffusion coefficients of grain boundary are more than five orders of magnitude higher than the bulk diffusion coefficients, suggesting that cation diffusion in grain boundaries may involve vacancy hopping between neighboring sites.^[^ ^36^ ^]^

Although cations have much lower diffusion coefficients than oxygen ions and protons (the later usually falls in 10^−3^ cm^2^ s^−1^ order of magnitude), cation diffusion rates are still higher than expected and can occur even at relatively low temperatures. For instance, in La_0.6_Sr_0.4_CoO_3_, achieving a low chemical cation diffusion coefficient of below 10^−23^ cm^2^ s^−1^ to supress cation diffusions requiring temperatures as low as 460 °C.^[^ ^34^ ^]^ Furthermore, altering the composition of the material's upper layers, which can significantly influence the catalytic performance of the air‐electrode, requires only a few cation jumps. Therefore, optimizing microstructure to prevent fast grain boundary diffusion may not be a promising approach. Further, the formation of secondary phases is expected to impact cation diffusion, making this an important area of study particularly in the context of designing and in situ exsolving nanoparticles over electrodes. However, there is limited knowledge regarding how the formation of heterogeneous structures in electrodes alters diffusion coefficients and activation energies. This remains a topic for further exploration in the future.

## 3 Surface Characterization Techniques

Due to the fact that R‐PCC works at elevated temperatures, oxidative, and humidified atmospheres, it becomes particularly crucial to perform in situ characterizations on air‐electrodes. Electrochemical characterizations are non‐destructive and remain the most frequently used method to understand surface evolutions. However, their results reflect combined factors and require physical models for the interpretation of such surface reactions, which may introduce additional errors. In contrast, advanced spectroscopic characterizations provide material surface signals directly. Based on the detection depth, the frequently used surface characterizations techniques include low‐ energy ion scattering (LEIS) for outmost atoms layer, X‐ray electron spectroscopy (XPS, 1–10 nm), and secondary ion scattering mass spectrometry (SIMS) for hundreds nanometers.^[^ ^39^ ^]^ Other characterization techniques including scanning electron microscopy (SEM), transmission electron microscopy (TEM), Raman, and Fourier‐transform infrared spectroscopy (FT‐IR) are also frequently used and their detecting thickness can be tailored by adjusting the beam energies. Here, we categorize the characterization techniques into in situ methods and surface‐sensitive ex situ techniques (**Table** **1**), then provide detailed descriptions and examples of each technique.

**Table 1:** Summary of surface characterization techniques.

Characterization technique	Information obtained	Detection depth	Working pressure	Working temperature	Refs.
Electrochemical Characterization	Electrochemical signals	Typically reflects bulk information but can also provide insights into surface properties, depending on the experimental design.	Low to high	Room temperature –1000 ^o^C	[6, 40]
FTIR	Adsorbed species, surface properties	Micrometers to hundreds of micrometers, depending on the IR source and sample	Low to high pressure available for designed reactors	Usually −269 –1000 ^o^C, depending on the reaction chamber	[41]
Raman	Material structure and bonding	Micrometers to hundreds of micrometers, depending on the selected laser	Low to high pressure available for designed reactors	Usually −269 –1000 °C, depending on the reaction chamber	[15, 42]
XPS	Cation valence, bonding, electronic structures	1‐10 nm	10^−6^ to 10^−9^ Torr for conventional XPS and can go up to ≈10 Torr for NAP‐XPS	Room temperature –800 ^o^C	[30, 43]
LEIS	Cations and anions	Outermost atomic layer	10^−8^–10^−10^ Torr	Room temperature	[44]
ToF‐SIMS	Cations and anions	1‐2 nm	10^−8^–10^−11^ Torr	Room temperature	[45]
SEM	Surface morphology, element type	Tens of nanometers to micrometers, depending on the selected accelerating voltages	10^−4^–10^−6^ Torr (high vacuum mode); 0.1–20 Torr (low vacuum mode)	Room temperature –800 ^o^C	[46]
TEM	Surface morphology, atomic information, lattice structure	Hunderds of nanometers, depending on the accelerating voltages	10^−7^–10^−9^ Torr	Room temperature –800 ^o^C	[47]

### 3.1 Electrochemical Characterizations

Electrochemical characterization results typically represent a combination of surface reactions and bulk information. However, they can provide detailed surface‐specific information depending on the experiment design and characteristic time of the electrochemical process. Investigating surface reactions independently requires the fabrication of thin‐film electrodes or the use of physical models. Electrochemical impedance spectroscopy (EIS) involves applying voltage perturbation across a wide range of time frequencies, analyzing the frequency‐dependent resistance offers insights into elementary reactions, as each reaction has a specific response time to the induced voltages. Additionally, kinetic studies, through altering conditions such as oxygen partial pressure, water partial pressure, and temperature, can help elucidate reaction steps. Initially, Adler et al. presented an equation (Equation 3) to describe non‐charge‐transfer processes on solid oxide fuel cell porous cathodes.^[^ ^48^ ^]^ They showed that polarization resistance is closely related to the surface exchange coefficient (k_chem_) and bulk diffusion coefficient (D_chem_). To obtain values for k_chem_ and D_chem_, kinetic investigation methods such as electrical conductivity relaxation (ECR), the mass relaxation method, and isotopic tracer exchange combined with secondary ion mass spectrometry (SIMS) characterizations are commonly used on dense samples. Among these methods, ECR holds advantages of high accuracy, low requirement for testing equipment, and easy operation. Similar to other measurements that rely on the property where the detected signal is proportional to defect concentrations, the mechanism behind ECR is the electroneutrality condition, electrons migrate to neutralize the sluggishly diffused oxygen ions or protons when there is a sudden change in the external oxygen partial pressure. This time‐depended migration process is detected by recording the material's conductivity relaxation behavior (Equation 4).^[^ ^3^, ^49^ ^]^

(3) $$R_{chem}=\left(\frac{RT}{2F^{2}}\right)\sqrt{\frac{\tau }{\left(1-\varepsilon \right)c_{v}D_{v}ar_{0}\left(\alpha_{f}+\alpha_{b}\right)}}$$

(4) $$\frac{c_{app}\left(t\right)-c_{app}\left(0\right)}{c_{app}\left(\infty \right)-c_{app}\left(0\right)}=\frac{\sigma_{app}\left(t\right)-\sigma_{app}\left(0\right)}{\sigma_{app}\left(\infty \right)-\sigma_{app}\left(0\right)}$$

The *c_app_* (*t*), *c_app_* (0), and $c_{app}\left(\infty \right)$ represent oxygen concentrations at t, initial, and equilibrium time. σ_*app*_(*t*), σ_*app*_(0), and $\sigma_{app}\left(\infty \right)$ represent the sample conductivity at specific t, initial, and equilibrium time. However, the measured k_chem_ from ECR only reflects the rate‐limiting steps within their reaction chains. The obtained values may differ between dense and porous samples due to variations in preparation temperatures and particle/grain sizes. Obtaining 𝑘_chem_ and 𝐷_chem_ using ECR on porous samples is challenging, as porous electrodes are characterized by inhomogeneous ORR kinetics, where oxygen vacancy gradients between the surface and bulk are affected by factors such as gas diffusion, applied bias, and electrode morphology. The actual experimental profiles are influenced not only by 𝑘_chem_ and 𝐷_chem_ but also significantly by the electrode morphology.^[^ ^48^, ^50^ ^]^ Recently, it was found combining electrochemical characterization with numerical simulations achieved precisely determining surface reaction kinetics, approaching the real electrode operation conditions.^[^ ^50^, ^51^ ^]^

Although these electrochemical methods offer insights into elementary reactions, it generally relies on the assumption that the physical properties of the electrode remain constant during these tests, which differs significantly from reality because the defect concentrations and conductivities are sensitive to external conditions.^[^ ^40a,b^ ^]^ The combination of isotope exchange and electrochemical characterizations offers a viable approach to addressing the aforementioned challenges.^[^ ^6^, ^40^ ^]^ In use of different mobility of isotopic elements, it becomes feasible to isolate the proton or oxygen‐related reactions without changing materials’ properties. For example, through switching between H_2_O and D_2_O, it was found the middle‐frequency resistance, which likely related to surface reactions, show its dominate role at lower temperatures, and adding pure proton conducting phases could effectively enhance middle‐frequency resistance and decrease the activation energy.^[^ ^6d^ ^]^ However, it should also be careful to perform H/D isotopic exchange experiments when the material is not fully hydrated, this is because the different mass of H and D, which results in the different vibration frequencies and affect the thermodynamics and reaction rates. On the other hand, depositing oxides can modify surface properties, thereby either enhancing or deteriorating electrochemical performance and influence step reactions. For instance, pulsed laser deposition (PLD) of monolayered SrO or the infiltration of basic oxides such as Li_2_O and CaO can shift the work function of the host oxide, leading to improved electrochemical performance.^[^ ^16^, ^52^ ^]^ However, covering thick insert oxides, for instance, depositing BaO over the air‐electrode Pr_1.75_Ba_0.25_NiO_4+δ_ surface slows down surface reactions, leading to increased low‐frequency resistance, suggesting that resistances within this frequency range are related to surface reaction kinetics.^[^ ^53^ ^]^

### 3.2 In situ Fourier‐Transform Infrared Spectroscopy

**Figure 4:**
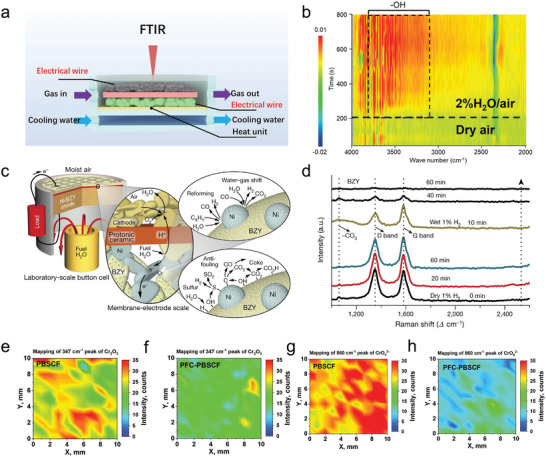
a) Schematic illustration of in situ FTIR test with R‐PCC in the reaction chamber. b) 2D diagram of FTIR results for PrBa_0.875_Cs_0.125_Co_2_O_5+δ_ tested at 300 °C by switching gas from dry air to 2%H_2_O/air at ≈200 s. Reproduced with permission.^[^ ^6d^ ^]^ Copyright 2024, John Wiley and Sons. c) Schematic illustration of PCFC and anodic water‐gas shift reaction, reforming of hydrocarbons, sulfur cleaning, and carbon cleaning processes. d) in situ Raman results of a carbon‐contaminated Ni‐BZY anode after exposing to various atmospheres. Reproduced with permission.^[^ ^42a^ ^]^ Copyright 2018, Springer Nature. Raman mapping of Cr_2_O_3_ (peak at 347 cm^−1^) on the e) PBSCF dense pellet and f) PFC‐PBSCF dense pellet after exposing to wet air with direct Cr alloy contact at 650 °C for 50 h; Raman mapping of CrO_4_^2−^ (peak at 860 cm^−1^) on the g, PBSCF dense pellet and h) the PFC‐PBSCF dense pellet after exposing to wet air with direct Cr alloy contact at 650 °C for 50 h. Reproduced with permission.^[^ ^42b^ ^]^ Copyright 2022, John Wiley and Sons.

In situ Fourier‐transform infrared spectroscopy (FTIR) is designed to detect material's structure or adsorbed species. FT‐IR using low energy infrared lights, different vibrations result from the specific species can be determined by analyzing wavenumber/ frequency. Since FT‐IR operates under ambient pressures, in situ tests can be performed on a reaction chamber with heating units, gas supplying system, and electrical units (**Figure** **4**a).^[^ ^6^, ^41^ ^]^

FT‐IR is frequently used to capture surface adsorbates, such as OH^−^, CO_3_^2−^, and C‐H species. Detecting basic or acidic surface species provide valuable information on a catalyst's acidity and basicity, which can offer insights into its activity for specific reactions. For example, in situ FT‐IR has been used to investigate CO_2_ and CO adsorption on Yttria‐stabilized zirconia (YSZ) and its components Y_2_O_3_ and ZrO_2_. The findings revealed that Y_2_O_3_ and YSZ more readily adsorb CO_2_ and CO than ZrO_2_, as evidenced by the formation of higher‐coordinated polydentate carbonates on YSZ and Y_2_O_3_ after CO_2_ adsorption. The basic hydroxyl species on the material surface are thought to be the adsorption sites for CO_2_. Additionally, in situ FT‐IR at elevated temperatures can help identify stable species or different adsorption configurations. For instance, when testing carbonate species on Y_2_O_3_ under 10 mbar CO_2_ and heating up to 873 K, it was observed that weakly adsorbed bicarbonate species desorbed with rising temperatures, leaving more strongly adsorbed species at higher temperatures. The order of carbonate species stability follows: monodentate bicarbonate → bidentate bicarbonate → bridged carbonate → polydentate carbonate.^[^ ^41b^ ^]^

Due to the critical role of water in R‐PCC air‐electrodes, its significant influence on electrode evolution and associated electrochemical performance has prompted some studies to investigate the behavior of surface water at elevated temperatures. Through comparative studies on cubic La_0.7_Sr_0.3_Mn_0.7_Ni_0.3_O_3‐δ_ (C‐LSMN) and rhombohedral La_0.7_Sr_0.3_Mn_0.7_Ni_0.3_O_3‐δ_ (R‐LSMN) materials using in situ FTIR, it was found the O‐H stretching is evident even at 600 °C for C‐LSMN, while disappeared for R‐LSMN when the temperature is higher than 400 °C. This proves that the cubic phase has stronger water adsorption ability and is more suitable for R‐PCC operations.^[^ ^41d^ ^]^ Zhang et al. employed in situ FTIR spectroscopy to investigate the variation in surface water and lattice proton signals of hydrated BaCo_0.7_(Ce_0.8_Y_0.2_)_0.3_O_3‐δ_ (BCCY) samples during thermal treatment from 50 to 750 °C. Their analysis revealed a marked increase in the intensity of the negative peak below 400 °C, followed by a more gradual rise at temperatures above 400 °C. This behavior indicates the existence of two distinct desorption processes, with surface water predominantly desorbing at temperatures below 400 °C, and lattice protons at temperatures exceeding 400 °C.^[^ ^41e^ ^]^ Besides, the researchers integrated the R‐PCC into the FTIR reaction chamber and connected it to an external electrochemical workstation. Humidified air or argon was introduced into the chamber, allowing real‐time detection of surface species via FTIR to investigate the correlation between surface protons and electrochemical current. Upon transitioning from dry air to 2% H₂O/air at 300 °C, hydroxyl species were immediately observed on the PrBa_0.875_Cs_0.125_Co_2_O_5+δ_ surface (Figure 4b), with their intensity increasing as the temperature decreased to 100 °C. Notably, the applied electrolysis current effectively consumed these adsorbed hydroxyl species, indicating that surface protons actively participate in the electrochemical process.^[^ ^6d^ ^]^

### 3.3 in situ Raman

When light is shed on a material, its photons interact with the electrons within the molecules. If inelastic collisions occur, a portion of the photon's energy is transferred to the electrons, causing the frequency of the scattered light to differ from that of the incident light. This type of scattering is called Raman scattering, and the resulting spectrum is the Raman spectrum. Raman can provide complementary information about materials structure and bonding on powder and bulk samples.^[^ ^54^ ^]^ Raman and FTIR are often complementary methods. Molecules with a symmetric center typically exhibit strong Raman activity and weak IR activity, whereas polar molecules show the opposite trend. The combination of these two techniques can generate more comprehensive information from investigated materials. FTIR is easier to perform and typically provides stronger signals, whereas Raman signals are relatively weaker, but its spectra rarely have overlapping bands, making spectral analysis more straightforward. FTIR is commonly used to study the asymmetric vibrations of polar groups, while Raman spectroscopy is often employed to investigate the symmetric vibrations of non‐polar groups. Raman spectroscopy can be used to analyze aqueous solutions, in contrast, infrared spectroscopy is unsuitable for analyzing aqueous solutions due to the strong absorption of water in the infrared region.

Because Raman tests are performed in ambient pressures, it is easy to construct reaction chamber for in situ characterizations. in situ Raman spectroscopy was utilized to study the mechanisms of carbon removal on the proton conductor BaZr_0.8_Y_0.2_O_3‐δ_ (BZY) to enhance PCFCs stability when directly operating on hydrocarbon fuels (Figure 4c,d). The findings revealed that carbon deposits remained stable under a dry reducing atmosphere. However, when switching to a wet reducing atmosphere, the carbon signal completely disappeared within 60 min. Furthermore, Raman analysis identified peaks corresponding to carbonate (‐CO_3_) species during the carbon removal process in wet hydrogen, indicating the involvement of carbonate intermediates in the carbon‐cleaning pathway.^[^ ^42a^ ^]^ Besides the active species, Raman also can be used to detect newly formed phases. Zhou et al. used Raman spectroscopy to verify the formation of BaCoO_3_ by treating the double perovskite PrBa_0.8_Ca_0.2_Co_2_O_5+δ_ under 40% H_2_O/air at 700 °C, BaCoO_3_ is identified at 614 cm⁻¹ and proved to be an active site for both the ORR and OER.^[^ ^15b^ ^]^ In addition, the mapping mode of the Raman tests enables determining the new phase produced at the specific area. Utilizing Raman to demonstrate PrBa_0.5_Sr_0.5_Co_1.5_Fe_0.5_O_5+δ_ coated by Pr_0.9_Fe_0.7_Co_0.3_O_3_ could improve cathode's stability under Cr contained atmospheres (Figure 4e–h). The possible impurities when encountered with Cr are Cr_2_O_3_ at 347 cm^−1^ and CrO_4_^2−^ at 860 cm^−1^. In contrast to pure PBSCF, the PFC‐PBSCF show low intensity of Cr_2_O_3_ and CrO_4_^2−^, suggesting that PFC catalyst is inert to Cr and has excellent stability.^[^ ^42b^ ^]^

### 3.4 X‐Ray Photoelectron Spectroscopy

X‐ray photoelectron spectroscopy (XPS) is commonly used to detect elements and electronic states in both powder and bulk samples.^[^ ^43a–f^ ^]^ It simultaneously measures the kinetic energy and emitted electrons within a range of 1 to 10 nanometers beneath the material's surface. Although X‐rays can penetrate several micrometers into the sample, the electron escape depth is much shallower with only ≈10 atomic layers. As a result, XPS primarily provides information about the material's surface. Conventionally, XPS operates under ultra‐high vacuum conditions to minimize collision losses of photoelectrons with residual gas molecules and to prevent the adsorption of these molecules on the sample surface. In order to simulate the reaction conditions, the XPS also allows for the pretreatment of samples under specific atmospheres, pressures, and temperatures before they are transferred to a high‐vacuum chamber for further XPS analysis. For example, it was expected doping fluorine into the perovskite Ba_0.5_Sr_0.5_Co_0.8_Fe_0.2_O_3‐δ_ oxygen site would decrease oxygen vacancy and only improves the material's stability. However, XPS results reveal that at elevated temperatures, the active oxygen species actually have higher ratio than that in undoped Ba_0.5_Sr_0.5_Co_0.8_Fe_0.2_O_3‐δ_ sample, implying a better catalytic performance for fluorine doped sample at intermediate temperatures.^[^ ^43g^ ^]^ It should be noted that assigning oxygen species is challenging, because the electrodes are highly active and can adsorb impurities during pretreatments. To improve the credibility of these assignments, additional experiments should be conducted by varying pretreatment conditions (*e.g*., *p*H₂O, *p*O₂) or referring other XPS peaks (e.g., C1s) to aid in the accurate identification of oxygen peaks.^[^ ^30^, ^55^ ^]^ By using ion etching (e.g., Ar⁺), *depth‐profiling* XPS can provide insights into the evolution of materials properties from the surface to the bulk. However, the high‐energy Ar⁺ beam may potentially alter the oxidation states of cations, posing additional challenges for accurate data interpretation. It was found that after steam corrosion on La_0.6_Sr_0.4_Co_0.2_Fe_0.8_O_3‐δ_, the material became enriched with La and Sr while being deficient in Co and Fe in a region ≈100 nm below the surface. This compositional change is proposed to be the cause of the decreased bulk diffusion coefficient and conductivity,^[^ ^56^ ^]^ and consistent with reported findings in other studies.^[^ ^57^ ^]^ In a case study of R‐PCC air‐electrodes, it was interpreted that the cation has slight oxidation after treating in wet air at intermediate temperatures when comparing to the bulk, yet the oxygen peaks shift to higher binding energies from surface to the bulk, these results indicate the surface undergoes oxidation reaction and the electrons may come from oxygen, which is different to the bulk proton uptakes that thought to reduce cations.

**Figure 5:**
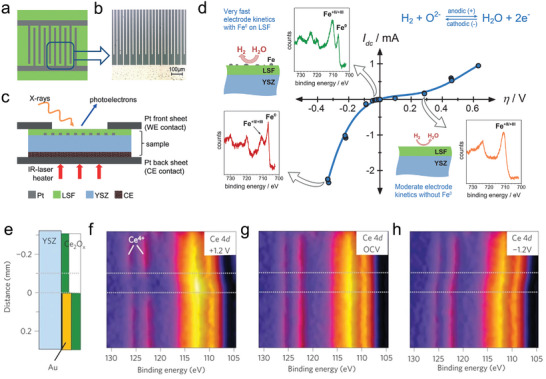
a) Schematic picture and b) optical picture of thin film LSF electrode and its current collector. c) Schematic picture of a sample for NAP‐XPS tests. d) Current‐overpotential curve of LSF that operated in humid reduction atmosphere (0.25 mbar H_2_‐0.25 mbar H_2_O). Reproduced with permission.^[^ ^43e^ ^]^ Copyright 2014, John Wiley and Sons. e–h) Distance‐resolved XPS spectra of the Ce 4*d* for the sample operated with +1.2, 0 and −1.2 V applied voltages. Reproduced with permission.^[^ ^43h^ ^]^ Copyright 2010, Springer Nature.

The development of key components in XPS has bridged the pressure gap, enabling XPS characterization across a wide pressure range from ultra‐high vacuum to tens of Torr or even higher. Near ambient pressure XPS (NAP‐XPS) allows for the investigation of materials in the presence of reaction gases, enabling the in situ capture of surface property evolutions.^[^ ^30^, ^43^ ^]^ For instance, NAP‐XPS was employed to investigate the role of hydroxylation on LaMO_3_ (M = Cr, Mn, Fe, Co, Ni) oxides in ORRs. The chamber was evacuated to a pressure of <1.5 × 10^−7^ Torr, and a water vapor pressure of 100 mTorr was introduced into the chamber. XPS results revealed lower *OH intensities on LaMnO_3_ and LaCoO_3_ compared to the other three films through analyzing O1s spectra. Furthermore, the ORR activity trends were found to inversely correlate with *OH coverage on LaMO_3_ films for aqueous oxygen electrocatalysis.^[^ ^43j^ ^]^ Zhang et al. performed NAP‐XPS for investigation of water effects to cation segregation in La_0.5_Sr_0.5_FeO_3‐δ_ electrode. The characterizations were performed at 650 °C with reaction gases of 200 mTorr O_2_ and variable 0—500 mTorr of H_2_O. They show the water vapor affects the strontium segregation weakly at low pressures but becomes dominate at higher pressures, and the presence of hydroxyl species may be the key to electrochemical performance degradations.^[^ ^30^ ^]^ The in situ XPS studies have directly proved that exsolved nanoparticles on perovskite oxides can enhance electrochemical performance. Opitz et al. used in situ NAP‐XPS to detect the evolution of surface species on a model electrode during water electrolysis (**Figure** **5**a–d). They mounted the sample between two platinum sheets to fix and achieve electrical contact between the electrodes. By analyzing the Fe3d peak, they observed an enhancement in electrochemical water splitting activity on the La_0.6_Sr_0.4_FeO_3‐δ_ (LSF) surface with the formation of Fe^0^ species, and this enhancement resulted in a highly asymmetric current‐voltage profile. However, the Fe^0^ species that induced by polarization voltage rapidly re‐oxidized upon the removal of polarization.^[^ ^43e^ ^]^ Furthermore, in situ NAP‐XPS can identify the active regions on the electrode surface. Zhang et al. investigated the oxidation state of an exposed surface using NAP‐XPS on single‐chamber cells consisting of CeO_2‐δ_/YSZ/Pt, operated at 750 °C with 1 mbar H_2_‐H_2_O as the reaction gas. By mapping the Ce^4^⁺ valence state under different voltages, they discovered that the electrochemically active regions extended ≈150 µm from the current collectors and were not confined to the three‐phase boundaries. These findings suggest that both surface reaction kinetics and electron transport are co‐limiting steps in the electrochemical process (Figure 5e–h).^[^ ^43h^ ^]^

### 3.5 in situ Microscopes

Scanning electron microscope (SEM) and transmission electron microscope (TEM) are probably most frequently used techniques to visualize materials surface. SEM uses focused electron beam to scanning sample's surface and collects signals (e.g. Secondary electron, backscattered electron, and X‐ray, Auger electron) then imaging material's surface. Limited by the operating principle and SEM equipment performance (e.g. electron gun, electromagnetic lens, and imaging system), the resolution is ranging from 1 to 100 nm. TEM uses accelerated electron beam to shed thin sample, then collecting the scattered electrons and the scattering angles, which reflects samples density and thickness. Due to the much higher accelerate voltages and shorter electron's De Broglie wavelength, the TEM can reach a very high resolution to 0.1 nm and able to image lattice structures. Many SEM and TEM equipped with energy dispersive spectroscopy (EDS) to precisely and quantitatively measure elements distributions and their respective ratios. Because of less accuracy in detecting light elements (e.g., oxygen, carbon etc.), some TEMs also equipped with electron energy loss spectroscopy (EELS) to enhance their application in light elements detections. SEM and TEM are widely used in R‐PCC and other fuel cell investigations. For instance, SEM was performed to investigate materials surface morphology change after annealing in steam,^[^ ^15^, ^29^, ^58^ ^]^ CO_2_,^[^ ^3^, ^59^ ^]^ or specific contamination.^[^ ^42^, ^60^ ^]^ In addition, composite oxides such as core‐shell structured, self‐assembled oxides and other oxides were developed and proved by TEM or SEM results.^[^ ^5^, ^10^, ^41^, ^61^ ^]^

**Figure 6:**
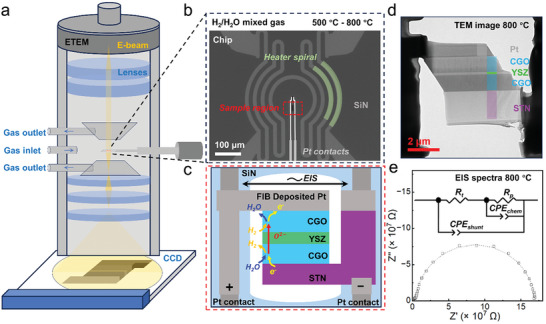
a) Schematic illustration of the environmental TEM and MEMS chip position; b) SEM image of the MEMS heating‐biasing chip; c) schematic diagram of the symmetric CGO‐YSZ‐CGO cell mounted at the sample region of the MEMS chip with Pt and STN current collectors of the cell connected to the Pt contacts of the chip for electrical polarization of the sample; d) TEM image of CGO‐YSZ‐CGO cell and e) corresponding EIS spectra recorded at temperature 800 °C. Reprinted with permission.^[^ ^47a^ ^]^ Copyright 2024, American Chemical Society.

Due to the low requirements to the operation pressures, it is possible to perform in situ observations on SEM and TEM. Shikazono's group use in situ SEM to investigate nickel migration in the anode, which is believed as the important cause to the anode deterioration.^[^ ^62^ ^]^ They demonstrated several steps for nickel migration and concluded that Ni migration occurs only at three‐phase‐boundary (TPB).^[^ ^46^ ^]^ Besides in situ SEM, Ma et al. showcased the feasibility of in situ TEM analysis on filmed solid oxide fuel cell/electrolysis cells (**Figure** **6**). The in situ TEM equipped with reaction gases, heating unites, and electrochemical characterization system.^[^ ^47a^ ^]^ Similarly, Jeangrous et al. performed in situ analysis of solid oxide fuel cell using environmental TEM to investigate the relationship of anodic nickel oxidation with catalytic activity toward H_2_ oxidations. They show as the O_2_ to H_2_ ratio increased to a certain value, the surface oxidation of Ni stopped fuel oxidation reaction, but the growth of Ni islands on the NiO restarts it.^[^ ^47b^ ^]^ in situ exsolution nanoparticles from oxides have demonstrated its broad application in catalysis and electrochemical devices, so that understanding the exsolution mechanisms becomes crucial. By conducting in situ TEM on a model catalyst of Ir‐doped SrTiO_3_ over different timescales, researchers observed the early stages of exsolution, host oxide evolution, and the socketing of exsolved Ir nanoparticles. It was found the surface defects such as Sr vacancy and host lattice restructuring trapped Ir atoms to form initial nanoparticles, which becomes the initial nucleation sites for further Ir cluster growth.^[^ ^47c^ ^]^

In most cases, the R‐PCC works with different reaction gases on its two electrodes, so that developing dual‐chambered reactor is crucial for better understanding the R‐PCC using in situ microscopes. However, to date, no reports have successfully conducted dual‐chamber tests for R‐PCCs at elevated temperatures with in situ TEM or in situ SEM, the major obstacle lies in the designing of the sample holder and gas supply system. More attentions are needed to pay on cell sealing and reaction chamber designs.

### 3.6 Low Energy Ion Scattering

**Figure 7:**
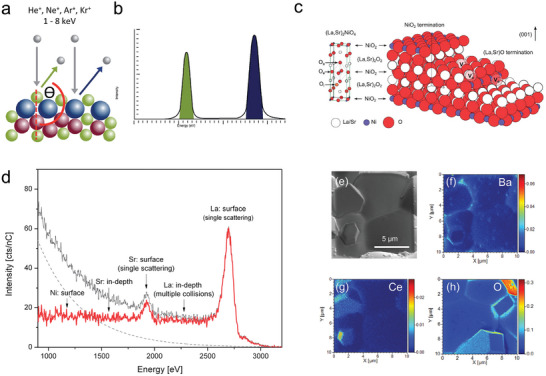
a) Noble gas ion bombard to the surface. b) the corresponding LEIS signal to the surface. Reprinted with permission.^[^ ^63^ ^]^ Copyright 2016, Royal Society of Chemistry. c) Schematic illustration of the (La,Sr)_2_NiO_4_, which consists of (La,Sr)_2_O_2_ bilayers and NiO_2_ monolayers along the c‐axis. d) ^22^Ne‐LEIS energy spectrum for the (001) face of the La_1.67_Sr_0.33_NiO_4+d_ single crystal that after heat‐treatment. Reprinted with permission.^[^ ^66^ ^]^ Copyright 2014, Royal Society of Chemistry. e) Surface SEM pictures of polished BaCeO_3_ pellets that was calcined at 1600 °C for 5 h. The TOF‐SIMS signal of f) Ba, g) Ce, and h) O elements at the polished surface. Reprinted with permission.^[^ ^45f^ ^]^ Copyright 2024, John Wiley and Sons.

Low energy ion scattering (LEIS) is a surface energy sensitive analytical technique that used in determining materials surface information with first layer atoms, such information contains surface structure, composition, and bonding. LEIS uses inert gas ions with specific energy to be incident on the sample surface and elastically collide with atoms on the sample surface. According to elastic scattering theory, the energy distribution of scattered ions is related to the atomic weight of surface atoms. By analyzing the scattered ion energy, information on the surface element composition and surface structure can be obtained (**Figure** **7**a,b). Because only ions are scattered off from the outermost atomic layers of the material, LEIS is uniquely sensitive to the atomic composition of the outermost layer of a material.^[^ ^63^ ^]^ This technique has been applied to the investigations of surface evolutions on air‐electrodes, such as cation segregation and grain boundary evolutions. Both bulk and powder samples are applicable for LEIS characterizations, yet the sample preparation should be performed carefully to avoid tiny surface pollution.

The LEIS experiments were frequently utilized on investigating cation segregation and cation migrations between neighborhood components.^[^ ^44c^ ^]^ Druce et al. studied the outer surface atoms in a series oxides include perovskite, double perovskites, and Ruddlesden‐Popper structured materials. A‐site cations segregation to the surface and B‐site cation rich layer behind surface were observed by performing LEIS characterizations on samples that treated at high temperatures, indicating such cation segregation should be a common phenomenon in a wide range electrodes.^[^ ^64^ ^]^ Similar results were obtained on double perovskites of LnBaCo_2_O_5+δ_ (Ln = Pr, Gd), LEIS results proves the B‐site cation (Co) is covered by the A‐site cation (Ba and Gd) after a long annealing time, the Ba segregation would disrupt the lattice order and will affect the surface exchange coefficients.^[^ ^44a^ ^]^ The Sr segregation also impede water incorporation process on proton conductor La_0.99_Sr_0.01_NbO_4‐δ_, which annealed at 1000 °C for 10 h, the LEIS experimental results estimated segregation layer can reach to 6–7 nm and impede water incorporation process due to elimination of oxygen vacancies.^[^ ^44b^ ^]^ Because the surface‐sensitive LEIS experiment can provide direct evidence of plane terminations, it enables to justify inconsistencies between theoretical calculations and experimental results. For example, through evaluating surface energy on La_2‐x_Sr_x_NiO_4+δ_ using DFT calculations, it was found the Ni‐contained surface are responsible for catalytic reactions and O^2−^ terminated (100) surface is the most stable surface on La_2_NiO_4+δ_ with NiO_6_ octahedra on the outermost plane.^[^ ^65^ ^]^ However, the LEIS with He^+^/3 keV and 22Ne^+^/5 keV beam energy shown the main peaks can be clearly attributed to the scattering of Sr and La elements, suggesting an inert surface on real air‐electrode (Figure 7c,d).^[^ ^66^ ^]^

### 3.7 Time‐of‐Flight Secondary Ion Mass Spectrometry

Secondary ion scattering mass spectrometry (SIMS) use focused ion beam (i.e., Xe, Ga, Cs, Ar) to sputter materials surface, and measuring the charge‐to‐mass ratio (m/z) of secondary ion that are ejected from material to analyze the near surface (1‐2 nm) elements. It is able to provide elemental information within two or three atomic layers of the surface. ToF‐SIMS is very sensitive, with limits of detection in the range of ppm‐ppb levels,^[^ ^67^ ^]^ however, the secondary ion yields can vary significantly within the same matrix among different components, or for the same component in different matrices, making absolute quantitative analysis challenging. Typically, the bulk‐shape samples are suitable for ToF‐SIMS experiments, and ToF‐SIMS is able to investigate the light elements such as oxygen and hydrogen, thus it is frequently used to measure the oxygen or proton surface exchange rate and bulk diffusion coefficients.^[^ ^45a–d^ ^]^

Surface water adsorption is particularly important for R‐PCC air‐electrodes because the dissociated protons are believed to enlarge the reaction zone then providing more reaction sites. Double perovskite oxide PrBaCo_2_O_5+δ_ is seen as an excellent candidate for oxygen transmission and catalytic reactions, shown great electrochemical performance in PCFC applications. However, ^18^O and ^2^H isotopic exchange combined with ToF‐SIMS experiments conclude ^2^H signals are nonhomogeneous and are likely associated with the presence of hydrated layers on the interior walls of the pores and not because of proton diffusion, suggesting this material has limited proton uptakes to its bulk.^[^ ^45e^ ^]^ On the other hand, it has been commonly believed that barium in proton conductors (e.g., BaZr_0.1_Ce_0.7_Y_0.1_Yb_0.1_O_3‐δ_ and BaZr_0.3_Ce_0.5_Y_0.2_O_3‐δ_) tends to evaporate during sintering at high temperatures. However, comparative studies using ToF‐SIMS on BaZrO_3_ and BaCeO_3_ have shown that barium is more likely to segregate to grain boundaries rather than evaporate. ToF‐SIMS results revealed a stronger Ba signal surrounding Ce‐rich segregated particles, indicating that the Ba aggregation at grain boundaries results from the Ce exsolution rather than thermal evaporation (Figure 7e–h).^[^ ^45f^ ^]^

## 4 Air‐Electrode Surface Composition Evolutions and Their Impacts on Electrochemical Performance

The air‐electrode of R‐PCCs undergoes proton‐involved ORR and OER. Unlike solid oxide fuel cells (SOFCs), where the ORR involves oxygen adsorption, charge transfer, oxygen dissociation, oxygen surface migration, and the incorporation of dissociated oxygen into oxygen vacancies.^[^ ^68^ ^]^ The ORR in PCFCs involve protons in stepwise reactions. In general, the reaction steps include a) oxygen adsorption, b) charge transfer, c) oxygen dissociation, d) proton diffusion, e) protons react with oxygen ions and form water, and f) water evaporation.^[^ ^40^, ^69^ ^]^ It should be noted that the protons can be provided by air‐electrode itself or by the triple‐phase boundaries, the step reactions may not require oxygen ion surface diffusion when protons are readily accessible,^[^ ^6b^ ^]^ step (e) also involves charge transfer and the presence of protons on the electrode surface can alter surface properties, thereby influencing the energy barriers of step reactions. On the other hand, the step reactions of OER at air‐electrode involve water adsorption, water dissociation combined with several charge transfer process, protons diffusion, and the association of oxygen ions to form oxygen molecules, which are then released into the atmosphere.^[^ ^40^, ^70^ ^]^ Many oxides have demonstrated the bulk or surface proton uptake abilities through hydration or hydrogenation reactions,^[^ ^6^, ^71^ ^]^ the presence of these protons can extend the reaction sites to the whole electrode and reduce activation energy.^[^ ^3b^ ^]^ However, excessive water adsorption also can increase polarization resistance, as the coverage of water molecules reduces the availability of active sites for O_2_ adsorption.^[^ ^40a^ ^]^ In general, the electrochemical performance of air‐electrodes is strongly influenced by their surface properties.

**Figure 8:**
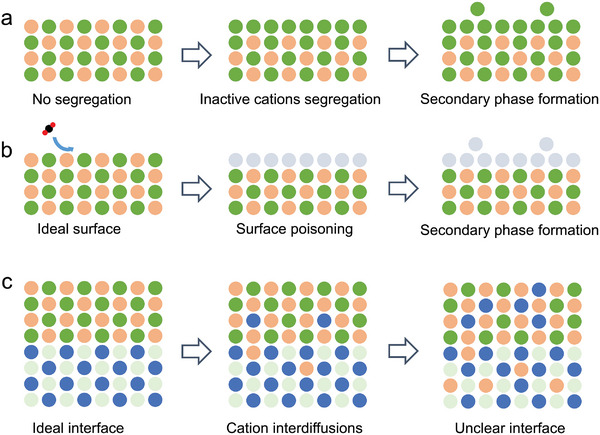
Schematic illustration of a) inactive cation surface segregation, b) surface poisoning, and c) cation interdiffusions.

The surface segregation of inactive cations, surface poisoning, and cation interdiffusions are major contributors to the deterioration of the air‐electrode surface (**Figure** **8**). These processes are influenced by materials properties, such as cation characteristics, defects, lattice strain, and electrostatics. Additionally, external condition, such as temperature, oxygen partial pressure, and reactive gases, have a strong impact. In this section, we discuss these phenomena, their formation mechanisms, and their influences on electrochemical performance. Special attention is given to the impact of water on these processes and, conversely, its contributions to performance enhancements.

### 4.1 Inactive Cation Surface Segregation

Cation surface segregation is a phase separation process that break the lattice symmetries, the exact driving force of cation segregation is still debatable. Among the discussed factors are kinetic demixing under thermal, electrical, and oxygen partial pressure gradients, it also affected by material intrinsic properties such as cations mismatch, non‐stoichiometric, and lattice strains.^[^ ^23^, ^34^, ^39^ ^]^ In the perovskite oxides with a formular of ABO_3_, cation segregation includes A‐site segregation and B‐site exsolution process, the former usually results in the electrochemical performance degradation even the segregation thickness is less than 20 nm, while the latter sometimes improves electrochemical performances. For instance, DFT calculations over CoO_2_ and SrO terminated surface in La_0.5_Sr_0.5_CoO_3‐δ_ suggest that the metastable CoO_2_ surface has 2–3 orders magnitudes faster ORR rate than SrO surface.^[^ ^72^ ^]^ Here, we summarize the main contributors to inactive cation segregations, including the material's intrinsic properties of lattice strain and surface electrostatic energy, and external reaction conditions including atmosphere, temperature, and applied voltages.

#### 4.1.1 Lattice Strain and Surface Electrostatic Energy Effects to Cation Segregation

The first contributor to cation segregation is lattice strain. In case of R‐PCCs, the air‐electrode naturally works in humidified atmospheres, and water uptake usually results in the lattice expansion due to the hydration reaction.^[^ ^73^ ^]^ For example, the expansion ratio of lattice constant is ≈0.15% for pure proton conductor BaZr_0.8_Y_0.2_O_3‐δ_ when switch from dry Ar to 3.12%H_2_O/Ar at 600 °C.^[^ ^73a^ ^]^ The air‐electrodes generally have a lower hydration ability than electrolytes, so the volume change caused by proton uptake should be small. However, air‐electrodes can undergo significant thermal and chemical expansions as temperature increases.^[^ ^74^ ^]^ Therefore, understanding of lattice strain to cation segregation becomes crucial in terms of R‐PCC air‐electrodes.^[^ ^75^ ^]^ In metal catalysts, changes in lattice constants alter the d‐band width, causing the d‐band center relative to the Fermi energy level to shift up or down, which results in modified chemical activity.^[^ ^76^ ^]^ In a perovskite, once the A‐site was dopped with other elements or experience water uptakes, it will trigger cation rearrangement and cation segregation in the process of annealing. It has been accepted that the elastic energy result from cation mismatching caused by doping different sized cations can be expressed as Equation (5).

(5) $$E_{elastic}=\frac{24\pi GKr_{a}r_{b}{\left(r_{a}-r_{b}\right)}^{2}}{3Kr_{a}+4Gr_{b}}$$

where K is solute's bulk modulus, G is the shear modulus of the solvent, *r_a_* and *r_b_* are the cation radii of solute and solvent.^[^ ^27^, ^77^ ^]^ However, this equation needs to satisfy two restrictions, first, it only applicable to diluted solutions, second, the lattice strain that result from the atom mismatch should be fully relaxed.^[^ ^27^ ^]^ Cai et al. using pulsed laser deposition (PLD) method to prepare La_0.8_Sr_0.2_CoO_3_ on the top of SrTiO_3_ and LaAlO_3_ substrates, with the purpose of introducing tensile strain and compressively strain by the small difference of substrates lattice parameters. They found both the tensile strained and compressively strained samples had Sr‐rich surfaces. Furthermore, such cation segregation caused by lattice strain also affects electron structures.^[^ ^76^, ^78^ ^]^ A transition from semiconductor to metal (SMT) were observed at a very low temperature of 200–300 °C, attributing to the interaction between oxygen vacancy and Co d‐band in a delocalized manner, and further be evidenced by the DFT calculation results that oxygen vacancy formation energy lowered by 0.43 eV on the 1.0% tensile strained sample to the −1.9% compressively strained one.^[^ ^79^ ^]^ Inspired by this, substituting A‐site cations with smaller radii cations would depress cation segregation, Lee et al. proves Ca dopped LaMnO_3_ material has most stable surface comparing to Ba or Sr doped LaMnO_3_ material, which is stable up to 830 °C for 1 h in air.^[^ ^27^ ^]^

Surface electrostatic energy also contributes to the cation segregation. Lee et al. reported that even though elastic energy is proposed to play an important role in controlling cation segregations, Ca, with a smaller radius than La, still segregates to the surface after long time annealing, implying that the elastic energy is not the sole factor to control cation segregations.^[^ ^27^ ^]^ Electrostatic energy resulting from charge interactions between dopants, oxygen vacancy, and polar surface is considered as another factor to drive cation segregations. Excess charge is commonly present on any electrode surface. For example, it has been predicted that the vacancy concentration at the surface of La_0.9_Sr_0.1_MnO_3‐δ_ under fuel cell operating conditions (*p*O_2_ = 1 atm at 1173 K) is ≈10^6^ times higher than that in the bulk.^[^ ^23^, ^27^ ^]^ The formation of oxygen vacancies leads to an electrostatic driving force between positively charged oxygen vacancies and negatively charged dopants ($Sr_{La}^{\prime }$, $Ba_{La}^{\prime }$, $Ca_{La}^{\prime }$), which result in the cation segregations. For example, DFT calculation results show that doping higher valence elements in Sr and B‐site, lower valence elements in La site, or create La vacancy in La_1‐x_Sr_x_Co_1‐y_Fe_y_O_3‐δ_ can suppress surface charge and Sr segregation.^[^ ^80^ ^]^ Tsvetkov et al. found that modifying the La_0.8_Sr_0.2_CoO_3‐δ_ surface with less reducible cations, such as Hf^4+^, Ti^4+^, Zr^4+^, Nb^5+^, Al^3+^ can increase the Co oxidation state on the surface and decrease oxygen vacancies, which lead to smaller electronic driving force for Sr segregation.^[^ ^81^ ^]^

#### 4.1.2 External Reaction Atmosphere, Temperature, and Applied Voltages to Cation Segregation

**Figure 9:**
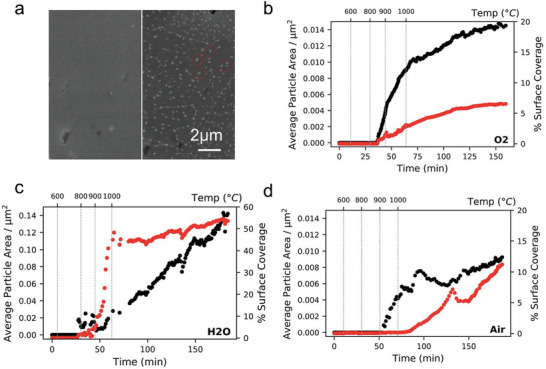
a) SEM pictures of the LSCF sample annealed in 3 mbar O_2_ at 624 °C (left hand) and 1000 °C (right hand), the formed particles are marked with red circles in the right‐hand picture. Higher temperature annealing induces secondary phase formation on the surface. Average particle area (black dots) and percent surface coverage (red dots) with respect to time and temperature for an LSCF sample annealed in b) 3 mbar oxygen, c) 3 mbar H_2_O and d) 3 mbar ambient lab air. Reprinted with permission.^[^ ^83a^ ^]^ Copyright 2018, Royal Society of Chemistry.

Phase segregation is thermodynamically governed by the reaction atmosphere and temperature. At sufficiently high temperatures, thermodynamic requirements are met, and the phase separation occurs with sufficient kinetics. Research results indicate Sr segregation to the surface can be observed even the temperature down to 400 °C and show an intensive segregation at higher temperatures.^[^ ^82^ ^]^ SrO segregation on La_0.6_Sr_0.4_Co_0.2_Fe_0.8_O_3‐δ_ (LSCF) surface was investigated as a function of temperatures and results indicate more segregated SrO nanoparticles when the temperature exceed 600 °C (**Figure** **9**a).^[^ ^83^ ^]^ Although higher temperatures kinetically favor cation diffusion and promotes segregated particles agglomerations, such particle coverage decreased at 800 °C than 700 °C, indicating the Ostwald ripening above 700 °C, leading to the vertical growth with bigger particles to decrease surface energy.^[^ ^83a^ ^]^ Oxygen partial pressure (*p*O_2_) is tightly related to the lattice nonstoichiometric and hence the lattice strains. Sr segregation was found to increase with increasing oxygen partial pressures and reach to the maximum at *p*O_2_ = 0.21 atm, but there is negligible SrO formation under pure N_2_ on LSCF cathode. Further, the SrO segregation phenomenon is also depressed when *p*O_2_ exceed 0.21 atm. Besides oxygen partial pressure, Niania et al. proposed the impurities including H_2_O or CO_2_ that contains in air induced SrO segregation, the rate of Sr segregation was found to be significantly accelerated in the presence of pure water compared to ambient air and pure O_2_ (Figure 9b–d). The particle growth was rapid from 850 to 1000 °C under pure water treatment, reaching a maximum surface coverage of 40%–50%. This is because the chemisorbed hydroxyl groups are proposed stay at the surface of the material, therefore, the segregated strontium will react with hydroxyl groups and form Sr(OH)_2_. However, the SrO is thermodynamically more stable at above 800 °C, therefore the particle growth at above 850 °C may occur after the decomposition of Sr(OH)_2_ into SrO.^[^ ^83a^ ^]^ Similar results were observed in other air‐electrodes such as La_0.5_Sr_0.5_FeO_3‐δ_, PrBa_0.5_Sr_0.5_Co_1.5_Fe_0.5_O_5+δ_, LaMnO_3_ based materials, and SrCo_0.95_Sb_0.05_O_3‐δ_ materials.^[^ ^29^, ^30^, ^31^ ^]^ The underlying mechanism for water promoted cation segregation may due to the thermodynamic favorable and alternation of surface defects, thus providing higher electrostatic attraction to the doped cations toward the surface.^[^ ^29a^ ^]^

In addition to temperature and atmosphere, external applied voltages also play crucial roles. The measured voltage in a solid oxide cell is directly related to the difference in the oxygen chemical potentials or the oxygen partial pressures ($U_{cell}=\left|\Delta \mu_{O_{2}}/nF\right|$) between the two electrodes according to the Nernst equation.^[^ ^84^ ^]^ Consequently, it is feasible to alter the oxygen partial pressure at the electrode by applying voltages. With negative voltages (cathodic polarization) applied on the cell, the reduction reactions occur on the air‐electrode, which may have similar effects to chemical reductions. For example, applying ‐0.25 V to the single cell depresses Sr surface segregation and form Co/Fe enriched surface on La_1‐x_Sr_x_Co_1‐x_Fe_x_O_3_‐Sm_0.2_Ce_0.8_O_1.9_ electrode.^[^ ^85^ ^]^ The CoO nanoparticles readily exsolve under electrochemical reductions with −2 V bias applied to Pr_0.2_Ba_0.8_Co_0.7_Fe_0.3_O_3‐δ_.^[^ ^86^ ^]^ Some works utilized this method to boost surface activity by strategically applying electrical bias. For instance, Zhang et al. performed electrochemical reductions on solid oxide fuel cells with Ln_0.2_Ba_0.8_Co_0.7_Fe_0.3_O_3‐δ_ (Ln = La, Pr, Nd). They found by applying a negative voltage of 2 V for 150 s, the CoO nanoparticles are exsolved and electrochemical performance was enhanced. The Pr_0.2_Ba_0.8_Co_0.7_Fe_0.3_O_3‐δ_ exhibits a low area specific resistance of 0.119 Ω cm^2^ at 550 °C, which is 1/3 of pristine sample.^[^ ^86^ ^]^

### 4.2 Air‐Electrode Poisoning

Air‐electrode poisoning begins with competitive adsorption between impurities and reactants. The slow desorption of these impurities hinders further reactions with the reactants. Additionally, these stably adsorbed species can lead to the formation of secondary phases that cover active sites required for step reactions, causing irreversible changes to the electrode surface. CO_2_ poisoning is the most encountered phenomenon because it is naturally present in ambient air (≈0.038 vol.%). Moreover, CO_2_ gas from the anode side, resulting from gas leaks when using hydrocarbon fuels, also contributes to CO_2_ poisoning. In addition to CO_2_, impurities such as chromium and silicon that originating from sealants or interconnection parts, also frequently result in cathode failure. Through analyzing EIS curves, it was observed that the low‐to‐middle frequency resistance associated with surface‐related reactions increases, suggesting that poisoning effects hinders surface adsorption and the formation of impurities obscured the surface active sites.^[^ ^87^ ^]^ The air‐electrode poisoning is a common phenomenon that happens on most state‐of‐art air‐electrodes such as La_0.6_Sr_0.4_Co_0.2_Fe_0.8_O_3‐δ_, Ba_0.5_Sr_0.5_Co_0.8_Fe_0.2_O_3‐δ_, Sm_0.5_Sr_0.5_CoO_3‐δ_, and PrBaCo_2_O_5+δ_, which are proved metastable in high CO_2_ concentrated atmospheres.^[^ ^7^, ^13^, ^88^ ^]^ For example, Ba_0.5_Sr_0.5_Co_0.8_Fe_0.2_O_3‐δ_ was found immediately stopped oxygen permeation by sweeping pure CO_2_ gas at 875 °C, attributing to the formation of dense carbonate layer with ≈5 µm and covers on the outer surface, preventing further reactions.^[^ ^89^ ^]^

#### 4.2.1 Factors Influence Surface Poisoning

Lewis's acid‐base theory can predict CO_2_ poisoning effects. As an acidic gas, CO_2_ is thermodynamically prone to adsorb on perovskites because alkaline cations typically at the A‐site. Sanderson's scale and the associated electronegativity equalization principle are effective in calculating bond energies and elucidating the acidic and basic properties of catalysts. Jeong et al. extended this method to lanthanides and other oxides, quantitatively scaling 101 metal oxides. They established the acidity order of frequently used oxides as BaO < SrO < CaO < MgO < ZrO_2_ < TiO_2_ < SnO_2_ < Ta_2_O_5_.^[^ ^16a^ ^]^ Doping with acidic cations is expected to enhance poisoning resistance. For example, it was found that SrSc_0.175_Nb_0.025_Co_0.8_O_3‐δ_ exhibits better stability than Ba_0.5_Sr_0.5_Co_0.8_Fe_0.2_O_3‐δ_ under CO_2_ atmospheres. The cation acidity follows the order of Nb^5+^ > Co^4+^ > Co^3+^ > Fe^3+^ > Sc^3+^ > Co^2+^ > Fe^2+^ > Sr^2+^ > Ba^2+^. Using Nb^5+^ and Sr^2+^ can enhance the CO_2_ resistance of SrSc_0.175_Nb_0.025_Co_0.8_O_3‐δ_ compared to Ba_0.5_Sr_0.5_Co_0.8_Fe_0.2_O_3‐δ_. Sc^3+^ exhibits higher acidity than Co^2+^ and Fe^2+^ while having greater basicity than Fe^3+^ and Co^3+^. At elevated temperatures, the valance state of Co and Fe tend to reduce to below 3+, so that Sc doping may also benefit for CO_2_ resistivity.^[^ ^87b^ ^]^ Similarly, Tejuca et al. investigated CO_2_ adsorption on LaBO_3_ (B = Cr, Fe, Co) over a wide temperature range and found the CO_2_ coverage follows the order of LaCrO_3_ > LaFeO_3_ > LaCoO_3_.^[^ ^90^ ^]^

Besides cations, the oxygen vacancies are frequently regarded as adsorption sites for CO_2_. The number of these vacancies increases with temperature, facilitating CO_2_ adsorption at higher temperatures (although excessively high temperatures can lead to CO_2_ desorption). In the Ba_0.5_Sr_0.5_Co_0.8_Fe_0.2_O_3‐δ_ material, the CO_2_‐temperature programmed desorption (TPD) peaks shift to higher temperatures, and the peak area, which reflects the amount of CO_2_ adsorption, also enlarges when Ba_0.5_Sr_0.5_Co_0.8_Fe_0.2_O_3‐δ_ is treated under CO_2_ gas at elevated temperatures. Furthermore, the calculated activation energy for desorption increases when the sample treated at higher temperatures, proving a stable adsorption configuration with the adsorbent.^[^ ^18c^ ^]^ On the contrary, filling these oxygen vacancies, either through controlling atmosphere or doping strategies, can alleviate CO_2_ poisoning. Yan et al. conducted a comparative study on Ba_0.5_Sr_0.5_Co_0.8_Fe_0.2_O_3‐δ_ material treated under CO_2_ and CO_2_‐O_2_ mixture gas, finding that the desorption peak for CO_2_ in the presence of O_2_ decreased by 29%–97% for adsorption temperatures ranging from 400 to 700 °C.^[^ ^18c^ ^]^

**Figure 10:**
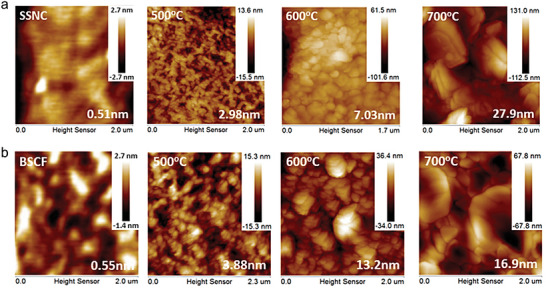
The AFM pictures of a) SrSc_0.175_Nb_0.025_Co_0.8_O_3‐δ_, b) Ba_0.5_Sr_0.5_Co_0.8_Fe_0.2_O_3‐δ_ surface before and after treatment in CO_2_ atmosphere between 500 and 700 °C. The “nm” denotes the roughness of surfaces. Reprinted with permission.^[^ ^87b^ ^]^ Copyright 2016, American Chemical Society.

Further increasing temperatures usually resulting in the formation of carbonates. Thermodynamically, the Ellingham diagram can be used to evaluate the stability of these carbonates. It indicates that BaCO_3_ forms more easily than SrCO_3_ and CaCO_3_, suggesting that doping Ba at the perovskite A‐site typically results in lower phase stability under CO_2_‐containing atmospheres.^[^ ^91^ ^]^ It was found that after treating SrSc_0.175_Nb_0.025_Co_0.8_O_3‐δ_ in CO_2_ atmospheres, orthorhombic structured carbonates with tiny grains and higher roughness are formed, whereas flower‐like carbonates are observed on the Ba_0.5_Sr_0.5_Co_0.8_Fe_0.2_O_3‐δ_ cathode (**Figure** **10**). CO_2_‐TPD and XRD results indicate that multiple carbonates observed on Ba_0.5_Sr_0.5_Co_0.8_Fe_0.2_O_3‐δ_ are Sr_x_Ba_1‐x_CO_3_ with different cation ratios (x) or the varying in crystallinity of impurities.^[^ ^87b^ ^]^ The actual formation of these carbonates is complex due to the kinetic diffusion of cations. The growth of BaCO_3_ follows a parabolic rate law over time in BaCo_0.4_Fe_0.4_Nb_0.2_O_3‐δ_ material, with increases in *p*CO_2_ or *p*O_2_ leading to faster membrane degradation. Kinetic studies have shown that the formation of the impurity layer is a diffusion‐controlled process, governed by the slow diffusion of Ba^2+^ from the bulk to the outer surface, and is driven by the chemical potential gradient of Ba^2+^ between the perovskite bulk and the gas‐solid interface.^[^ ^7a^ ^]^

#### 4.2.2 Water Influence on Electrode Poisoning

The presence of water may exacerbate CO_2_ poisoning. Studies have shown that more oxygen vacancies may be generated in the presence of water at material surface, and these oxygen vacancies play a critical role in CO_2_ adsorption and bicarbonate formation.^[^ ^18^, ^29^ ^]^ Additionally, water can accelerate surface cation segregation, leading to the formation of alkaline oxides that readily react with CO_2_ to form carbonates.^[^ ^29^, ^30^, ^31^ ^]^ However, the practical analysis presents challenging because hydroxyl species that formed over oxides can be either acidic or basic depends on the adsorption site and the crystallinity of the oxides.^[^ ^41^, ^92^ ^]^ In addition, the presence of water also affects oxides lattice structures, causing lattice expansion and distortion, bring more uncertainties when analyzing such water effects to the degradation. Besides material's intrinsic properties, the poisoning situation may be different in case of successive H_2_O and CO_2_ adsorption, or the co‐adsorption of H_2_O and CO_2_ at the same time. Yi et al. reported that the oxygen permeation flux through Sr_0.95_Co_0.8_Fe_0.2_O_3‐δ_ decreased dramatically when the air stream contain both H_2_O and CO_2_, while such degradation is slow when contain either CO_2_ or H_2_O solely.^[^ ^18a^ ^]^ Zhao et al. fond the surface carbonates on LSCF after treating in CO_2_ atmospheres at the temperature range between 400–680 °C and the presence of water vapor aggravates the reaction between CO_2_ and LSCF.^[^ ^18b^ ^]^ Yan et al. systematically investigated the co‐adsorption of CO_2_ and O_2_ in the presence of H_2_O on BSCF at various temperatures using TPD measurement. They found the CO_2_ desorption temperature is slightly higher and the peak area is larger in the presence of H_2_O, indicating the CO_2_ poisoning become severe when encounter with water. Through comparing the CO_2_ desorption peak after the samples were pretreated under various conditions, it was found that the desorption peak area decreases following the order of CO_2_> CO_2_‐O_2_‐H_2_O>CO_2_‐O_2_, proving the presence of water aggravate the CO_2_ poisoning effect.^[^ ^18c^ ^]^ It is also proposed that such degradation in the presence of both H_2_O and CO_2_ resulting from the formation of bicarbonate that covers the cathode surface and the oxygen vacancy plays essential role in this process. However, experiment results also indicate high temperatures (>810 °C) thermodynamically unfavorable for the formation of such bicarbonates due to the decreased formation entropy and enthalpy, as well as unfavored water uptake effects for air‐electrodes.^[^ ^18^, ^93^ ^]^

### 4.3 Cation Interdiffusion Between Neighborhood Components

Cations often experience interdiffusions between neighboring components during the fabrication of R‐PCCs at high temperatures or during testing at evaluation temperatures. Although air‐electrode sintering temperatures are much lower than those required for electrolyte densification, air‐electrode materials rich in transition metal elements and nanoscale particles exhibit higher reactivity and can readily react with the electrolyte at even lower temperatures. In general, materials with different configurations tend to react with each other when the reaction Gibbs free energy is less than zero, which is driven by changes in enthalpy or entropy and provides the driving force for cation interdiffusion. Besides thermodynamic effects, the extent of interdiffusion is also influenced by cation mobility and diffusion regions, with grain boundary diffusion being orders of magnitude faster than diffusion within the grain interior.^[^ ^94^ ^]^

**Figure 11:**
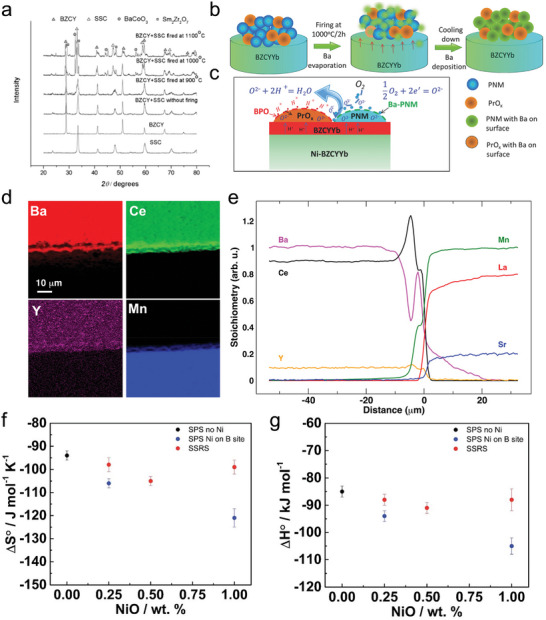
a) XRD patterns of BaZr_0.1_Ce_0.7_Y_0.2_O_3‐δ_, Sm_0.5_Sr_0.5_CoO_3‐δ_, and BaZr_0.1_Ce_0.7_Y_0.2_O_3‐δ_‐Sm_0.5_Sr_0.5_CoO_3‐δ_ mixtures before and after calcinations at 900, 1000, and 1100 °C for 3 h. Reprinted with permission.^[^ ^20b^ ^]^ Copyright 2008, John Wiley and Sons. b) Hypothesis of formation of Ba coating on hybrid cathode surface. c) schematic of electrode reaction processes. Reprinted with permission.^[^ ^21c^ ^]^ Copyright 2017, John Wiley and Sons. d) LSM/BCY annealed at 1150 °C for 72 h. Concentration maps of barium (red), cerium (green), yttrium (violet), manganese (blue). e) Concentration profiles of barium (pink), cerium (black), yttrium (orange), manganese (green), lanthanum (red), and strontium (blue). Reprinted with permission.^[^ ^98^ ^]^ Copyright 2019 American Chemical Society. f) The standard hydration enthalpy and g) hydration entropy of samples with bulk Ni, Ni‐free BZCY samples synthesized using the spark plasma sintering (SPS) method, and samples prepared via the solid‐state reactive sintering (SSRS) approach. Reprinted with permission.^[^ ^102^ ^]^ Copyright 2021, Royal Society of Chemistry.

Cation interdiffusion often involving one or more cations, this process can result in the formation of new phases that may either degrade electrochemical performance or, alternatively, create phases with higher catalytic activity or the ability to concurrently conduct protons, oxygen ions, and electrons, thereby enhancing electrochemical performance.^[^ ^20^, ^21^, ^95^ ^]^ The co‐sintering of BaCe_0.9_Y_0.1_O_2.95_ and Ba_0.5_Sr_0.5_Co_0.8_Fe_0.2_O_3‐δ_ leads to Ba diffusion from the electrolyte into the electrode. EIS results indicate that such cation diffusion does not significantly alter the polarization resistance but contributes substantially to an increase in ohmic resistance, likely due to the formation of an insulating layer at the electrode/electrolyte interface.^[^ ^20a^ ^]^ In some cases, cation diffusion between the electrolyte and electrode can result in the formation of catalytically active phases or proton‐conducting phases. For instance, the co‐sintering of BaZr_0.1_Ce_0.7_Y_0.2_O_3‐δ_ and Sm_0.5_Sr_0.5_CoO_3‐δ_ leads to the formation of new phases of BaCoO_3_ and Sm_2_Zr_2_O_7_ (**Figure** **11**a). The BaCoO_3_‐based phase is known as an excellent mixed ionic and electronic conductor with high catalytic performance and conductivity, while Sm_2_Zr_2_O_7_ is a proton conductor. Electrolyte‐electrode assemblies sintered at 1000 °C show lower polarization resistance compared to those sintered at 900 or 1100 °C.^[^ ^20b^ ^]^ Similarly, barium diffusion from BaZr_0.1_Ce_0.7_Y_0.1_Yb_0.1_O_3‐δ_ electrolyte into the composite air‐electrode PrNi_0.5_Mn_0.5_O_3_‐PrO_x_ was observed after sintering at 1000 °C for 2 h. This process produced a proton‐conducting BaPrO_3_ phase and a Ba‐doped PrNi_0.5_Mn_0.5_O_3_ phase with high oxygen vacancy concentration coating on the air‐electrode surface, significantly enhancing the electrochemical performance (Figure 11b,c).^[^ ^21c^ ^]^

Investigating such cation interdiffusion is challenging for several reasons. One major challenge is that these processes occur over thousands of hours at operating temperatures, and mimicking such reactions requires higher temperatures over tens or hundreds of hours. Additionally, the techniques used to investigate interdiffusion are often complementary but have limitations. For example, XRD characterization requires large sample volumes and yields average values, while micro‐XAS provides space‐resolved data but can vary based on sample geometry and preparation, making it difficult to obtain consistent results.^[^ ^94^ ^]^

#### 4.3.1 Cation Diffusion from Electrolyte to Air‐Electrode

Cations inside the electrolyte can diffuse to air‐electrode upon annealing, Lin et al. performed interdiffusion studies on the mixture of Ba_0.5_Sr_0.5_Co_0.8_Fe_0.2_O_3‐δ_ – BaCe_0.9_Y_0.1_O_2.95_ with mass ratio of 1:1 and sintering from 900 to 1100 °C for 2 h with 50 °C intervals. Their XRD results show the BSCF (110) peak shifts to lower angles with increasing sintering temperatures, while the BCY (211) peak almost unmoved, implying reactions between BSCF and BCY happened. Considering that the BSCF can endure a large deviation of its A/B cation ratio from 0.85<A/B<1.09 but with obvious lattice change,^[^ ^96^ ^]^ while the BCY doesn't show such obvious lattice change even it also able to permit large A/B deviations,^[^ ^97^ ^]^ it is likely that barium inside BCY electrolyte diffuse to BSCF then form Ba‐deficient in BCY and Ba‐enrichment in BSCF.^[^ ^20a^ ^]^ In the subsequent O_2_‐TPD analysis, the β peak which corresponds to Co^3+^ reduction to Co^2+^, disappear on samples that treated at higher temperatures, corresponds to another work that Ba enrichment in BSCF results in the absence of β peak.^[^ ^96c^ ^]^ These results prove the co‐sintering of electrode and electrolyte likely result in the cation diffusions from electrolyte to electrode.

#### 4.3.2 Cation Diffusion from Air‐Electrode to Electrolyte

The active cations inside the air‐electrode also possible to diffuse into electrolyte when annealing the sample at elevated temperatures. Through annealing the La_0.8_Sr_0.2_MnO_3_‐BaCe_0.9_Y_0.1_O_3_ diffusion couple at 1150 °C for 72 h, a Ba‐ and Mn‐rich layer with a peculiar shape, consisting of small arcs 7–10 µm wide, was observed (Figure 11d). Manganese diffuses significantly up to ≈3 µm beyond the interface and in small amounts as far as 18 µm. Element concentration profiles from BaCe_0.9_Y_0.1_O_3_ to La_0.8_Sr_0.2_MnO_3_ indicate a Ce‐ and Y‐rich phase was identified at the interface (0 µm), a Ba‐ and Mn‐rich phase was found at −1 µm, and the depletion of Ba and an increase in Ce give rise to another Ce‐ and Y‐rich phase at −5 µm (Figure 11e).^[^ ^98^ ^]^ Generally, cation diffusion can extend over a very long distance when annealed at high temperatures. It was reported the depth of cobalt diffusion from PrBaCo_2_O_5+δ_ into 1 wt.% NiO‐doped BaZr_0.8_Y_0.2_O_3‐δ_ can reach 1000 µm after annealing at 1200 °C for 48 h in air. The high annealing temperature may also cause PrBaCo_2_O_5+δ_ to melt, likely contributing to the extensive diffusion range of cobalt.^[^ ^99^ ^]^ It was reported crystal structures have important influence on interdiffusions. Chiara et al. conducted structure compatibility between Ba_1‐x_La_x_FeO_3‐δ_ and oxygen ion conductor Gd_0.2_Ce_0.8_O_2‐δ_ or proton conductor BaZr_0.825_Y_0.175_O_3‐δ_ in use of XRD and micro X‐ray Fluorescence (XRF). Ba_0.95_La_0.05_FeO_3‐δ_ and Ba_0.85_La_0.15_FeO_3‐δ_ show higher activity than Ba_0.5_La_0.5_FeO_3‐δ_, which may result from their lager deviation of Goldschmidt factor from lattice unity and lower stability of perovskite structure, providing a larger enthalpic driving force for the interfacial reaction. Moreover, a significant reaction is observed with proton conductor BaZr_0.825_Y_0.175_O_3‐δ_, forming Fe_x_O_y_, La_2_O_3_, La_2_Zr_2_O_7_, and (Ba, La)(Fe, Zr, Y)O_3‐δ_ impurities. However, in terms of GDC, the majority of impurity phase contains Fe_x_O_y_ and Gd doped BaCeO_3_.^[^ ^94^ ^]^

The cation diffusion from air‐electrode to electrolyte has pronounce effects to electrolyte's properties. Wan et al. performed studies on the cobalt substation to the cerium in BaZr_0.1_Ce_0.7_Y_0.1_Yb_0.1_O_3‐δ_, it was found the cobalt incorporation into the electrolyte decreases BZCYYb's conductivities, and increase the proton conducting activation energy, implying the protons were more readily trapped and difficult to jump between neighbor oxygen atoms.^[^ ^100^ ^]^ Huang et al. conducted detailed studies on the nickel diffusion to the water uptake abilities of proton conductor Ba(Zr, Ce, Y)O_3‐δ_ through performing TG tests. They show the proton source decreased significantly, for example, the 1 wt.% NiO (correspond to 4.0 at% on the B‐site) decreased proton uptake by one half. Such phenomenon resulted from the decreased effective acceptor concentration and formation of (Ba, Ni, Y)O_x_ liquid phase at grain boundaries, leading to the unavailable of BaO to the perovskite A‐site. The Ba deficiency also result in the partial change of Y from being an acceptor on the B‐site to the doner on the A site, decreasing the effective acceptor concentration.^[^ ^101^ ^]^ Figure 11f,g show the hydration enthalpies (Δ*H* ^0^) and entropies (Δ*S* ^0^) for the samples without Ni or with Ni in the B site, and with excessive NiO as extracted from liner Van't Hoff plots. The B‐site doping with Ni result in more negative value for Δ*H* ^0^ and Δ*S* ^0^, which possibly ascribe to the proton trapping at doubly, negatively charged $Ni_{Zr}^{,}$ defects.^[^ ^102^ ^]^ However, some reports indicate the incorporation of transition metal elements into the proton conducting electrolytes enhanced the phase stability under harsh conditions, finding that incorporating Fe and Ni_0.5_Fe_0._ into BaZr_0.1_Ce_0.7_Y_0.2_O_3‐δ_ electrolytes show improved stability under CO_2_ contained atmospheres without detection of secondary phases.^[^ ^103^ ^]^

Up to now, many works trying to alleviate such cation interdiffusion effects by engineering the electrolyte/electrode interface or decreasing the co‐sintering temperatures. Previous reports indicate the cation diffusion mainly occurs at temperatures higher 900 °C, thus decreasing cathode sintering temperature as low as 900 °C and concurrently fabricating cathode slurry with nanosized particles, which enables the tight bonding between cathode and electrolyte, can ensure the high electrochemical performance and avoid cation diffusions. Another commonly used strategy is adding a proton conducting buffer layer to stop the cation diffusion between electrolyte and electrode. It was found through adding a porous buffer layer of BaZr_0.1_Ce_0.7_Y_0.1_Yb_0.1_O_3‐δ_ between BaZr_0.8_Yb_0.2_O_3‐δ_ and La_0.6_Ba_0.4_CoO_3‐δ_, slightly increased the ohmic resistance and deteriorates output performance, it enhanced the stability by stopping the cobalt diffusion from air‐electrode to the electrolyte, for example, the degradation rate decreases from 4.8% to 0.9% kh^−1^, and the PCFC demonstrate the low degradation rate of 0.8% kh^−1^ for 3000 h.^[^ ^104^ ^]^

### 4.4 The Water Impacts on R‐PCC Air‐Electrode Reaction and Induces Active Phase Formations

#### 4.4.1 Water Uptakes

The water in air‐electrode can either from external humid atmosphere when performing OER reactions or released from ORR reaction (Equations 1 and 2), addressing the water uptake ability on R‐PCC air‐electrodes since these oxides with proton conducting property can enlarge the reaction zone to the whole electrode.^[^ ^6b^ ^]^ Unlike electrolytes, where water uptake follows a hydration reaction with water molecules filling oxygen vacancies and forming two proton defects (Equation 6), the p‐type material with abundant electron holes also undergoes a hydrogenation reaction (Equation 7), which involves electron holes and lattice oxygen. It was found that materials with higher basicity generally exhibit decreased Gibbs free energy for proton uptake. The basicity of perovskite oxides can be defined by the electronegativity of the cations (χ_*ion*_) and is represented by the equation $\chi_{ion}=z_{ion}\;/r_{ion}^{2}$, where *z_ion_* is the ion's charge and *r_ion_* is the Shannon ion radius.^[^ ^6^, ^105^ ^]^ In Zohourian's work, a decreasing trend in proton uptake was observed with increasing p‐type electronic conductivities in cobalt/zinc‐containing perovskites. The proposed underlying mechanism is that factors favoring the delocalization and high mobility of p‐type carriers (such as increasing B‐O covalency, low oxygen vacancy concentration, and the presence of redox‐inactive cations in perovskites) simultaneously decrease the basicity of oxides.^[^ ^6a^ ^]^

(6) $$H_{2}O+O_{O}^{X}+V_{O}^{\cdot \cdot }\to 2{\mathrm{OH}}_{O}^{\cdot }$$

(7) $$H_{2}O+2O_{O}^{X}+2h^{\cdot }\to 2{\mathrm{OH}}_{O}^{\cdot }+1/2O_{2}$$

In contrast to bulk proton incorporation, the formation of surface protons through water adsorption is a common characteristic observed in many oxides.^[^ ^43^, ^55^, ^106^ ^]^ These surface protons are believed to be capable of transporting along the material's surface and grain boundaries.^[^ ^107^ ^]^ Notably, an unexpected anti‐Arrhenius behavior has been observed in their conductivities at temperatures below 300 °C, where conductivity increases as temperature decreases due to the enrichment of surface protons under lower temperatures.^[^ ^71^ ^]^ The surface proton uptake can be directly proved using FTIR and its proton transport mechanisms were further revealed using H/D isotopic exchange experiments. It was found the proton transport mechanism change from Grotthuss type to vehicular transport when the temperature and humidity exceed to a certain value, correspondently, the surface water layer change from an “ice‐layer” to “water‐layer”.^[^ ^108^ ^]^ Raz et al. proposed the proton hopping mechanism on Y_2_O_3_‐doped ZrO_2_ oxide as the water dissociation and subsequently disproportionation reaction.^[^ ^109^ ^]^ Sato et al. proposed that proton migration on the ZrO_2_ surface occurs through two mechanisms, direct proton transfer from Zr‐OH₂ to Zr‐OH⁻ and a chain of protonation and deprotonation reactions involving surrounding H₂O molecules. This suggests that proton conduction involves the coexistence of Zr‐OH_2_ and Zr‐OH⁻, facilitating the transfer of protons between Brønsted acid and base sites.^[^ ^110^ ^]^

#### 4.4.2 Water Impacts to Electrochemical Performance

The presence of protons directly influences electrochemical performance, as evidenced by experimental results showing that surface protons can be regulated through external voltages, as demonstrated by in‐situ FTIR characterizations on the PrBa_0.875_Cs_0.125_Co_2_O_5+δ_ air‐electrode, and constructing hydrophilic air‐electrode by fabricating PrBa_0.875_Cs_0.125_Co_2_O_5+δ_‐BaZr_0.1_Ce_0.7_Y_0.1_Yb_0.1_O_3‐δ_ composite electrode further improves surface reactions.^[^ ^6d^ ^]^ Wang et al. studied the proton effects to ORR in hydrogenated BaZr_0.75_Co_0.25_O_3‐δ_ (100) surface with four proton defects and one oxygen molecule adsorbed on the surface using DFT calculations. The calculated reaction heat and energy barrier indicate that the migration of two protons to an oxygen molecule that adsorbed on an oxygen vacancy to break the O─O bond is the most feasible process, indicating the presence of protons on the catalyst surface facilitates the oxygen dissociation reaction.^[^ ^6c^ ^]^ Similar results were obtained in Ruddlesden‐Popper structured Sr_3_Fe_2_O_3‐δ_ oxide, where the oxygen dissociation reaction energy barrier reduce from 2.28 to 1.46–1.58 eV with the presence of surface protons. It is worth noting that the slab with protons is likely more reduced than the proton‐free slab, which could also contribute to the reduced O_2_ dissociation energy barrier.^[^ ^19a^ ^]^ However, too much water adsorption result in slow ORRs. He et al. investigated polarization resistance with different water contents on Sm_0.5_Sr_0.5_CoO_3‐δ_‐BaZr_0.3_Ce_0.5_Y_0.2_O_3‐δ_ air‐electrode, they found the polarization resistance increases with water content from 20% to 70% in open circuit voltage, probably due to the inhibited oxygen adsorptions caused by the water molecular coverage. However, such polarization resistance have different trend under 1.3 V electrolysis voltage when performing PCEC.^[^ ^40a^ ^]^ Similar results were obtained in other works, the authors demonstrated the low frequency resistance decreased with vapor concentration, indicating that high steam concentration facilitate gas diffusion and improve electrochemical performance, but too high vapor concentration will filling oxygen vacancies with hydroxyl species in BaZr_0.1_Ce_0.7_Y_0.1_Yb_0.1_O_3‐δ_ electrolyte and inhibit proton conductivity, contributing to the degradation of electrochemical performance.^[^ ^5e^ ^]^

#### 4.4.3 Water Induces the Formation of Active Phase

**Figure 12:**
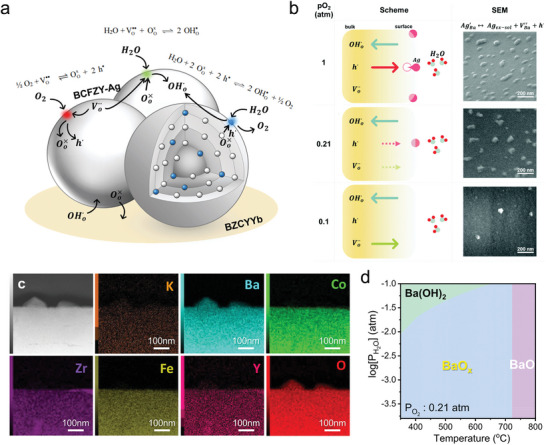
a) Illustration of water‐induced exsolution on triple conductors. b) Relation of water‐mediated exsolution and hydrogenation reaction with 1 vol% H_2_O mixed with different *p*O_2_, the right side show their corresponding SEM pictures of BCFZY‐Ag depending on *p*O_2_. Reprinted with permission.^[^ ^19b^ ^]^ Copyright 2022, Royal Society of Chemistry. c) EDS mapping results of KBCFZY with BaO_4_ nanoparticles exsolved on the outside. d) Thermochemical calculation results of Ba(OH)_2_ and BaO_x_ compositions as functions of temperature and water concentration (*p*H_2_O). Reprinted with permission.^[^ ^111^ ^]^ Copyright 2024, Royal Society of Chemistry.

In some cases, the presence of water induces surface evolutions, leading to the formation of active nanoparticles on the air‐electrode surface and enhances electrochemical performance. Unlike proton‐conducting electrolytes that typically undergo a hydration reaction to incorporate protons, the hydrogenation reaction (Equation 7) which consumes electronic holes, is a reduction process. When these electronic holes originate from cations, it is possible to reduce the cations to a lower valence state, thereby driving the exsolution process. Kim et al. utilized this method to in situ exsolve silver nanoparticles from the BaCo_0.4_Fe_0.4_Zr_0.1_Y_0.1_O_3‐δ_. They found that steam induced silver exsolution when the sample was treated under high *p*O_2_ atmospheres, while these Ag nanoparticles almost absence under lower *p*O_2_ because the low *p*O_2_ inhibits the hole generation and materials has low conductivities (**Figure** **12**a,b). The silver nanoparticle exsolution not only enhanced surface reaction but also improved bulk diffusion coefficients. Consequently, the peak power density of the fuel cell increased by 1.47 times to 1.2 W cm^−2^ at 650 °C.^[^ ^19b^ ^]^ Park et al. proposed that the hydrogenation reaction might not be the dominant process in the exsolution mechanism, because during the formation of cation vacancies, the generated holes are often consumed through recombination with the abundant electrons that present in highly electron‐conductive electrode materials. Instead, they suggest that the exsolution of BaO_x_ from K_0.05_Ba_0.95_Co_0.4_Fe_0.4_Zr_0.18_Y_0.02_O_3‐δ_ under a water atmosphere is primarily due to the high volatility of barium. When exposed to high water vapor, barium spontaneously forms Ba(OH)_2_ under high vapor pressures, which then thermodynamically converts to BaO_x_ (Figure 12c,d). Additionally, the relaxation of lattice strain caused by the difference in ionic radii of doped cations, along with the chemical stability influenced by defect interactions, also contributes to the exsolution of barium.^[^ ^111^ ^]^

The interpretation of water‐promoted exsolution becomes more complicated when exsolved nanoparticles fall into the oxide range. Zhou et al. found a hexagonal phase BaCoO_3‐δ_ can be formed on PrBa_0.8_Ca_0.2_Co_2_O_5+δ_ after annealing in 40% H_2_O/air at 700 °C. Such BaCoO_3‐δ_ nanoparticles activates the electrode surface and reduces the polarization loss in both fuel cell mode and electrolysis mode.^[^ ^15b^ ^]^ Similarly, the water‐induced Nb‐deficient PrBaCo_1.6_Fe_0.2_Nb_0.2‐x_O_5+δ_ are observed on PrBaCo_1.6_Fe_0.2_Nb_0.2_O_5+δ_ oxides, which facilitates the surface exchange process and enhances electrochemical performance.^[^ ^15c^ ^]^ In another example, the water‐promoted Nb‐deficient Ba_0.9_Co_0.7_Fe_0.2_Nb_0.1_O_3‐δ_ nanoparticle formations on Ba_0.9_Co_0.7_Fe_0.2_Nb_0.1_O_3‐δ_. The authors invested the formation mechanisms by performing segregation energy calculations for co‐doped Ba(B_0.5_B’_0.5_)O_3_ (B, B’ = Co, Fe, and Nb) and show the segregation energy follows the order of Nb>Fe>Co, indicating that Co and Fe are more likely to segregate from the bulk to the surface than Nb and leading to the formation of Nb‐deficient nanoparticles.^[^ ^15a^ ^]^ However, the exact driving force for such exsolution process remain for further studies.

## 5 Strategies to Activate/Stabilize Air‐Electrode Surface

The development of air‐electrodes has made significant progress in recent decades (**Table** **2**). Initially, precious metals such as platinum (Pt) were used.^[^ ^112^ ^]^ However, these materials generally exhibited low electrochemical performance due to the lack of oxygen conduction pathways. Later, mixed oxygen ion and electron conductors (MIECs), such as Ba_0.5_Sr_0.5_Co_0.8_Fe_0.2_O_3‐δ_,^[^ ^20a^ ^]^ La_0.6_Sr_0.4_Co_0.2_Fe_0.8_O_3‐δ_,^[^ ^113^ ^]^ PrBaCo_2_O_5+δ_,^[^ ^114^ ^]^ and Sm_0.5_Sr_0.5_CoO_3‐δ_ were introduced.^[^ ^40a^ ^]^ These materials were used either as single phases or as composites with electrolyte materials to enhance proton diffusion channels. Their application significantly improved electrochemical performance while reducing costs. In the past decade, the development of triple conducting air‐electrode has become a hot research topic.^[^ ^3^, ^6^ ^]^ A series of triple conductors capable of conducting oxygen ions, electrons, and protons have been developed. Air‐electrodes such as BaCo_0.4_Fe_0.4_Zr_0.1_Y_0.1_O_3‐δ_ and PrBa_0.5_Sr_0.5_Co_1.5_Fe_0.5_O_5+δ_ can expand the reaction zone across the whole electrode and enhancing the electrochemical performance of R‐PCCs, particularly at lower temperatures. However, these air‐electrodes still often suffer from performance degradation due to surface evolution. Here, we present strategies and examples of research efforts aimed at stabilizing the electrode surface and enhancing catalytic activity.

**Table 2:** Performance of R‐PCCs with single phase air‐electrode or electrode/electrolyte composite air‐electrodes at 600 °C.

Cell configuration [fuel‐electrode|electrolyte|air‐electrode]	Polarization resistance at open circuit voltages [Ωcm^2^]	Open circurt voltage [V]	Peak power density [mWcm^2^]	Current density at 1.3 V [mA cm^−2^] and air‐electrode side reaction gas	Year	Refs.
Pt|BCY25|Pt	–	1.1	70	–	2002	[112a]
Pt|BCY10|Pt	–	1.0	–	12 (ambient air)	2008	[112b]
Ni‐BCY|BCY10|Ba_0.5_Sr_0.5_Co_0.8_Fe_0.2_O_3‐δ_	≈0.1	∼1.02	400	–	2008	[20a]
Ni‐BZCY172|BZCY172|PrBaCo_2_O_5+δ_	0.92	1.03	148	–	2009	[114]
Ni‐BZCY532|BZCY532|BZCY532‐Sm_0.5_Sr_0.5_CoO_3‐δ_	2.41	0.97	69	∼200 (50%H_2_O/air)	2010	[40a]
Ni‐BZCYYb1711|BZCYYb1711|BaCo_0.4_Fe_0.4_Zr_0.1_Y_0.1_O_3‐δ_	0.07	1.1	650	∼1000 (20%H_2_O/air)	2015	[3, 115]
Ni‐BZY|BZY|BZY‐Sr_2_Fe_1.5_Mo_0.5_O_6‐δ_	0.48	0.86 (with 10%H_2_/N_2_ in fuel‐electrode)	94	210 (3%H_2_O/air)	2017	[116]
Ni‐BZCYYb4411|BZCYYb4411| PrBa_0.5_Sr_0.5_Co_1.5_Fe_0.5_O_5+δ_	0.14	1.0	1098	1920 (3%H_2_O/air)	2018	[3, 117]
Ni‐BZCY262|BZCY262|BZCY262‐Pr_2_NiO_4+δ_	0.78	0.98	–	400 (40%H_2_O/air)	2018	[70a]
Ni‐SZCY|SZCY| Ba_0.5_La_0.5_CoO_3‐δ_	1.7	0.75 (with 1% H_2_/Ar in fuel‐electrode)	–	200 (1%O_2_‐80% H_2_O/Ar)	2018	[118]
Ni‐BZCY172|BZCY172| SrEu_2_Fe_1.8_Co_0.2_O_7‐δ_‐BZCY172	0.89	0.99	220	400 (10%H_2_O/air)	2018	[5c]
Ni‐BZCY721|BZCY721|Ba_0.7_Gd_0.8_La_0.5_Co_2_O_6‐δ_ (Tubular cell)	3.8	0.9	–	∼100 (50% H_2_O, 0.03% O_2_‐ 47.3% Ar, 3 bar)	2019	[119]
Ni‐BZCYYb4411|BZCYYb4411|Pr_0.5_Ni_0.5_CoO_3‐δ_	0.3	1.06	528	∼1000 (10%H_2_O/air)	2020	[120]
Ni‐BZCYYb1711|BZCYYb1711| La_0.6_Sr_0.4_Co_0.2_Fe_0.8_O_3‐δ_	0.39	1.07	650	∼1500 (3%H_2_O/air)	2021	[113]
Ni‐BZCYYb1711|BZCYYb1711| Pr_0.2_Ba_0.2_Sr_0.2_La_0.2_Ca_0.2_CoO_3‐δ_	0.26	1.08	720	1750 (3%H_2_O/air)	2023	[121]
Ni‐BZCYYb1711|BZCYYb1711| PrBa_0.9_Cs_0.1_Co_2_O_5+δ_	0.3	1.06	1190	1480 (3%H_2_O/air)	2023	[122]
Ni‐BZCYYb1711|BZCYYb1711| Nd_0.8_Sr_1.2_Ni_0.7_Fe_0.3_O_4±δ_	0.18	1.05	440	1540 (30%H_2_O/air)	2024	[123]
Ni‐BZCYYb1711|BZCYYb1711|Ba_0.95_La_0.05_Fe_0.8_Zn_0.2_O_3‐δ_	0.32	1.1	370	410 (10%H_2_O/air)	2025	[124]

BCY25: BaCe_0.75_Y_0.25_O_3‐δ_; BCY10: BaCe_0.9_Y_0.1_O_3‐δ_; BZCY172: BaZr_0.1_Ce_0.7_Y_0.2_O_3‐δ_; BZCY532: BaZr_0.5_Ce_0.3_Y_0.2_O_3‐δ_; BZCYYb1711: BaZr_0.1_Ce_0.7_Y_0.1_Yb_0.1_O_3‐δ_; BZY: BaZr_0.8_Y_0.2_O_3‐δ_; BZCYYb4411: BaZr_0.4_Ce_0.4_Y_0.1_Yb_0.1_O_3‐δ_; BZCY262: BaZr_0.2_Ce_0.6_Y_0.2_O_3‐δ_; SZCY: SrZr_0.5_Ce_0.4_Y_0.1_O_3‐δ_; BZCY721: BaZr_0.7_Ce_0.2_Y_0.1_O_3‐δ_.

### 5.1 Engineering Surface using Doping Strategy

The doping strategy is one of the most frequently used approaches to stabilize the electrode surface. The oxygen vacancy is usually seen as the reaction site to catalytic reactions, it also proved as the active site for CO_2_ adsorption and poisoning reactions, as well as the unstable sites that probably triggers inactive cation segregations, thus moderately decreasing oxygen vacancies and strengthen metal‐oxygen bond are expected to improve oxides stability.^[^ ^87^, ^88^ ^]^ Doping elements with high valance states, such as Nb^5+^, Ti^4+^, Mo^5+^, W^5+^, Y^3+^, Sb^3+^, Ta^5+^, would decrease oxygen vacancy based on electroneutrality condition.^[^ ^7^ ^]^ The electronegativity and the ionic size also need to be considered comprehensively, especially for the A‐site elements of perovskite.^[^ ^91^, ^125^ ^]^ For instance, it was found to replace 10% barium with higher electronegative lanthanum in Ba_0.5_Sr_0.5_Co_0.8_Fe_0.2_O_3‐δ_ and formulated as La_0.1_Ba_0.4_Sr_0.5_Co_0.8_Fe_0.2_O_3‐δ_ reduces basicity and improves the chemical stability in acid atmospheres. Moreover, the smaller La than Ba alleviates the lattice distortion and strengthens the bonds between La and lattice oxygen, which is also proposed as an important reason to the enhanced electrochemical performance.^[^ ^126^ ^]^ The stability of perovskite oxides can be reflected by the measure of bonding energy in crystalline ionic compounds. Hu et al. calculated the average metal‐oxygen bond energy (ABE) in SrCo_1‐x_Sb_x_O_3‐δ_ system according to: ABE = $\frac{1}{12}\;\left(\Delta H_{SrO}-\Delta H_{Sr}-\frac{1}{2}D_{O_{2}}\right)+\frac{1-x}{18}\left(\Delta H_{Co_{3}O_{4}}-3\Delta H_{Co}-\frac{4}{2}D_{O_{2}}\right)+\frac{x}{12}\left(\Delta H_{Sb_{2}O_{5}}-2\Delta H_{Sb}-\frac{5}{2}D_{O_{2}}\right)$. Where Δ*H_SrO_*, $\Delta H_{Co_{3}O_{4}}$, $\Delta H_{Sb_{2}O_{5}}$ are heat formation of these oxides, Δ*H_Sr_*, Δ*H_Co_*, Δ*H_Sb_* are heats of sublimation of metals of Sr, Co, and Sb, respectively, and $D_{O_{2}}$ is the dissociation energy of oxygen molecule. Calculation results show the ABE decreases (more negative) with increasing Sb contents, indicating gradual enhancements of structure stability and CO_2_ tolerance.^[^ ^7d^ ^]^ Although evaluating bonding energy from thermodynamic viewpoint provides a feasible way to predict oxides stability, such date usually not available for oxygen‐deficient oxides, and the lattice energy calculation also requires the knowledge of electron/coordination configuration and valance state of the B‐site cations, which are vary depending on the temperatures and oxygen partial pressures.^[^ ^7a^ ^]^ For example, the bond energy of Zr‐O (760 kJ mol^−1^) is smaller than that of Ce‐O (795 kJ mol^−1^), however, the bond energy cannot explain the improved stability of BaZrO_3_ than BaCeO_3_ under CO_2_ atmospheres.^[^ ^73^, ^127^ ^]^

**Figure 13:**
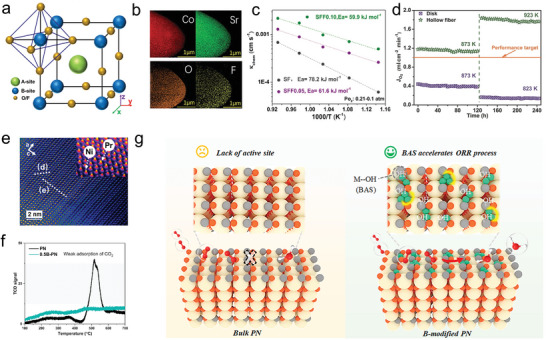
a) schematic illustration of perovskite with oxygen site occupied by oxygen atoms or fluorine. Reprinted with permission.^[^ ^131^ ^]^ Copyright 2016, John Wiley and Sons. b) EDS mappings of SrCoO_2.85‐δ_F_0.15_ sample. Reprinted with permission.^[^ ^129^ ^]^ Copyright 2019, Royal Society of Chemistry. c) *K*_chem._ for the SrFeO_3‐δ_, SrFeO_3‐δ_F_0.05_, and SrFeO_3‐δ_F_0.1_ from 600 to 800 °C. Reprinted with permission.^[^ ^130^ ^]^ Copyright 2017, John Wiley and Sons. d) long‐term stability of SrCo_0.9_Nb_0.1_O_3‐δ_F_0.1_ membranes with disk or hollow fiber shapes for oxygen permeation at various temperatures. Reprinted with permission.^[^ ^131^ ^]^ Copyright 2016, John Wiley and Sons. e) HAADF‐STEM images for 0.5B‐PN powders, the inset picture shows the energy intensity distribution of atoms. f) CO_2_‐TPD profiles of PN and 0.5B‐PN samples. g) Schematic diagram of activating PN surface with presence of B. Reprinted with permission.^[^ ^135^ ^]^ Copyright 2023, American Chemical Society.

Recently, the anion doping is emerged as the new strategy to stabilize perovskite surface^[^ ^43^, ^128^ ^]^ and phase structures^[^ ^129^ ^]^ (**Figure** **13**a,b). The anions with higher electronegativity such as fluorine (4.0) can attract more electrons from the nearby cations than the electronegativity of oxygen (3.44). If fluorine is doped into the perovskite oxygen sites, it is expected to reduce the valance electron density of oxygen ion, weaken the chemical bonds between cations and oxygen ion, and form more oxygen ion transporting paths. The ECR conducted on SrFeO_3‐δ_ (SF), SrFeO_3‐δ_F_0.05_ (SFF0.05), and SrFeO_3‐δ_F_0.1_ (SFF0.1) shown that F^−^ doping significantly enhanced the bulk diffusion and surface exchange properties of perovskites, the surface exchange coefficients of SFF0.05 and SFF0.1 are nearly one order of magnitude higher than those of SF (Figure 13c).^[^ ^130^ ^]^ In another example, experimental results have shown that XPS O1s peak shifts from 528.9 to 529.2 eV, and the O_2_ desorption peak in the O_2_‐TPD profile shifted from 680 K to 620 K upon incorporating F^−^ into the SrCo_0.9_Nb_0.1_O_3‐δ_F_0.1_ (SCNF) lattice, proving that oxygen ions more easily escape from the lattice. Furthermore, SCNF exhibited stable oxygen permeation over a period of 250 h (Figure 13d), and XRD results confirmed that SCNF maintained its perovskite structure after long‐term operation.^[^ ^131^ ^]^ However, incorporating F⁻ into oxygen sites reduces the oxygen vacancy concentration, which in turn decreases the proton concentration,^[^ ^132^ ^]^ this reduction may not be favorable for expanding the reaction zones in air‐electrode.

The heteroatoms, such as phosphorus (P) and boron (B), were also proved to efficiently optimize the oxide material's properties including conductivity, sintering ability, acidities, and structural stability. The P‐doped oxide La_0.5_Sr_0.5_Fe_0.9_P_0.1_O_3‐δ_ exhibits a single phase without detectable impurities but shows decreased lattice parameters due to the smaller ionic radius of P compared to Fe.^[^ ^133^ ^]^ The incorporation of B into La_0.6_Sr_0.4_Co_0.76_Fe_0.19_B_0.05_O_3‐δ_ expands lattice, likely because B doping reduces Co/Fe oxidation states, which have larger ionic radii than their higher valance state ions.^[^ ^134^ ^]^ In another example, a small amount of B doping (0.5 at%) into Pr_4_Ni_3_O_10+δ_ (PN) does not alter the lattice parameters, suggesting that B is primarily concentrated on the surface of Pr_4_Ni_3_O_10+δ_.^[^ ^135^ ^]^ Such surface modification can enhance CO_2_ resistance (Figure 13e,f), water uptake ability, and catalytic activity toward ORR. Attributing to the raising surface Bronsted acid concentration and depressing of the surface Lewis acidity (Figure 13g). DFT calculation results also proved that incorporating boron into the material reduces oxygen vacancy formation energy and O_2_ dissociation energy.^[^ ^135^ ^]^

### 5.2 Developing Oxides Beyond Perovskite

**Figure 14:**
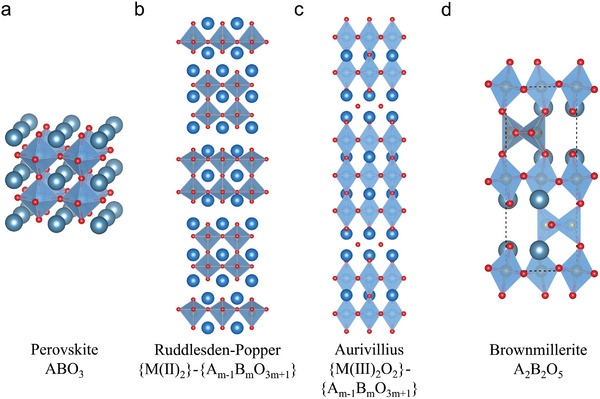
Schematic illustration of a) perovskite, b) Ruddlesden‐Popper oxide, c) Aurivillius oxide, d) Brownmillerite oxide.

Perovskite refers to both the mineral CaTiO_3_, which named after Russian mineralogist Lev Alekseevich Perovski, and the broader class of materials that share the CaTiO₃ (ABO_3_) crystal structure. A wide range of atomic species can satisfy Goldschmidt tolerance factor and Pauling's rules, contributing to the remarkable chemical flexibility of the perovskite crystal structure. Beyond the simple perovskite structure, the perovskite family also includes complex variants such as Ruddlesden‐Popper (RP) structured oxides and Aireville's oxides (**Figure** **14**).^[^ ^136^ ^]^

The RP perovskite is named after S. N. Ruddlesden and P. Popper, who conducted initial studies on Sr_n+1_Ti_n_O_3n+1_.^[^ ^137^ ^]^ The RP structure consists of rock salt layers and perovskite layers across crystallographic c‐axis direction and is usually formulated as [M(II)_2_]‐[A_m‐1_B_m_O_3m+1_] (M(II) is typically alkaline earth). As the m increases, the structure becomes less stable and prone to form perovskite.^[^ ^137a^ ^]^ The key for RP oxides to stabilize itself under harsh conditions lies in the R‐P oxides do not necessarily contain alkaline‐earth elements in A‐site, which alleviate them from fatal inert cation segregations. Druce et al. utilized surface sensitive LEIS to investigate the surface cation segregation on LSCF and PrLaNiO_4+δ_ (PLNO), and found that in contrast to LSCF, there is no lanthanide cation segregation to the surface on PLNO surface. The active oxygens inside the RP oxides can exist either as interstitial oxygen or oxygen vacancy depending on the valance states of the involved cations. Many researches have been reported using these oxides as R‐PCC air‐electrodes.^[^ ^8a–f^ ^]^ The substitution of La by Ca in La_2_NiO_4+δ_ and formulated as La_1.9_Ca_0.1_NiO_4+δ_ increases its conductivity and surface exchange coefficient at intermediate temperatures, these results indicate more oxygen are inserted into the lattice and form interstitial oxygen species.^[^ ^8d^ ^]^ Besides monolayered R‐P oxides, the double layered Sr_3_Fe_2_O_7‐δ_ was developed and utilized on PCFC air‐electrodes, it delivers extremely low oxygen vacancy formation energy and considerable oxygen diffusion energy barrier, as well as higher surface exchange coefficient than La_2_NiO_4+δ_.^[^ ^138^ ^]^ Further, the R‐P structured materials usually demonstrate promising proton uptake ability.^[^ ^139^ ^]^ According to DFT calculations, Sr_3_Fe_2_O_7‐δ_ (SFO) reduces the oxygen dissociation energy barrier in the presence of protons. Although proton migration across the rock‐salt layer is challenging due to the large distance, SFO provides an abundant proton source and facilitates easy proton conduction within the perovskite layer. The combination of improved surface reaction kinetics and rapid proton diffusion within the perovskite layer suggests that SFO is more suitable for PCFCs than for SOFCs.^[^ ^19^, ^140^ ^]^ Huan et al. co‐doped Eu and Co into SFO, resulting in the SrEu_2_Fe_1.8_Co_0.2_O_7‐δ_. It was observed that Sr atoms occupy the center of the perovskite slabs, while Eu atoms arrange orderly within the rock‐salt layer. This unique structure significantly reduces the Lewis basicity, enhancing resistance to water‐containing atmospheres. When this material was assembled as an air‐electrode in a PCFC, it demonstrated high electrochemical performance and stability in both fuel cell and electrolysis cell modes.^[^ ^5c^ ^]^

Aurivillius oxides contain perovskite layers and rock‐salt layers with the formular of [M(III)_2_O_2_]‐[A_m‐1_B_m_O_3m+1_], where the M(III) is typically bismuth. These oxides have attracted extensive studies in fast oxygen ion conductors, low‐fatigue ferroelectrics, and photocatalysts.^[^ ^141^ ^]^ It was found that Aurivillius Bi_2_Sr_2_Nb_2_MnO_12‐δ_ oxide that has n perovskite layers [A_n‐1_B_n_O_3n+1_]^2−^ and bismuth‐oxygen sheets [Bi_2_O_2_]^2+^, showing both great catalytic performance and stability in CO_2_ contained atmospheres. The oxide keeps stable even in 10 vol% CO_2_/air under heating and cooling and no phase transformations or the formation of new phases are detected. The protective rock‐salt layers may be the reason for such high stability. Besides, this cathode delivers low area specific resistance of 0.26 Ω cm^2^ at 750 °C and maintains stable even after subjecting such oxide into high‐concentrated CO_2_ for 5 h.^[^ ^142^ ^]^ Due the structural stability of this kind oxides, Shao et al. utilized Bi_2_V_0.9_Cu_0.1_O_5.35_ as air‐electrode in single‐chamber fuel cells with C_3_H_8_‐O_2_ mixture as reaction gas and demonstrate an out‐put performance of 60 mW cm^−2^ at 562 °C.^[^ ^8g^ ^]^ Brownmillerite oxides have a general formula of A₂B₂O₅, characterized by 1/6 of the oxygen sites being vacant. These intrinsic oxygen vacancies are fully ordered in rows along the [110] direction, resulting in a structure where corner‐sharing octahedral perovskite layers alternate with tetrahedral layers. Similar to RP oxides, the Brownmillerite oxides do not necessarily contain Sr or Ba cations inside the A‐site. It was found that Ca_2_FeO_5_ and Ca_2_Fe_1.95_Mg_0.05_O_5_ can be used as air‐electrode, the Ca_2_FeO_5_‐ Ce_0.9_Gd_0.1_O_1.95_ composite air‐electrode delivers a low area specific resistance (ASR) of 0.294 Ω cm^2^ at 700 °C.^[^ ^143^ ^]^

### 5.3 Constructing Core‐Shell Structured Air‐Electrodes

Core‐shell structured cathodes have been developed to stabilize and activate the electrode surface. These designs typically feature a shell with higher activity or stability to enhance surface performance, while the inner core provides oxygen, electron, and proton conduction paths.^[^ ^9^, ^144^ ^]^ In many cases, the shell is developed using advanced methods such as infiltration, electrospinning, or atomic layer deposition,^[^ ^9^, ^144^, ^145^ ^]^ the strategic in situ formation of core‐shell nanoparticles also reported in some studies.^[^ ^146^ ^]^ The addition of robust and active phases using infiltration methods is a common approach in many studies. For instance, directly infiltrating Co(NO_3_)_2_ solution onto the proton conductor BaZr_0.4_Ce_0.4_Y_0.2_O_3‐δ_ (BZCY) scaffold enhanced the activity and durability of cathode, attributing to the small size of nanoparticls caused by low temperature sintering, and similar thermal expansion coefficient (TEC) between dense BZCY and infiltrated BZCY, ensuring a good thermo‐mechanical compatibility.^[^ ^144b^ ^]^ Similarly, it was found that infiltrating PrBa_0.8_Ca_0.2_Co_2_O_5+δ_ into the classic LSCF cathode formated a comprised phases containing BaCoO_3‐δ_, PrCoO_3‐δ_, and conformal PrBa_0.8_Ca_0.2_Co_2_O_5+δ_ after co‐sintering. DFT calculations revealed that BaCoO_3‐δ_ actually exhibits higher activity than PrBa_0.8_Ca_0.2_Co_2_O_5+δ_, and the improved ORR kinetics are attributed to the enriched surface oxygen vacancies and rapid oxygen transport on PrBa_0.8_Ca_0.2_Co_2_O_5+δ_.^[^ ^9a^ ^]^ Infiltrating Sr(NO_3_)_2_ onto SrFe_0.9_Nb_0.1_O_3‐δ_ formed a Sr_3_Fe_1.8_Nb_0.2_O_7‐δ_ shell coating outside, which supressed the Sr segregation and enhanced the activity toward proton‐involved ORR.^[^ ^147^ ^]^ Similiarly, infiltrating A‐site deficient La_0.95_CoO_3‐δ_ onto the porous LSCF scaffold supressed Sr segregation and prolong the lifetime of cathode.^[^ ^145b^ ^]^ Besides supressing Sr segregations, the construction of core‐shell nano electrodes also improved the stability under harsh atmospheres. La_0.8_Sr_0.2_MnO_3‐δ_ coated Ba_0.5_Sr_0.5_Co_0.8_Fe_0.2_O_3‐δ_ was proved with enhanced stability under CO_2_ contained atmospheres, and demonstrated stable operation for over 300 min in 10 vol%‐CO_2_ containing mix gas.^[^ ^144c^ ^]^ Similarly, SrCo_0.8_Nb_0.1_Ta_0.1_O_3‐δ_ coated with La_2_NiO_4_ were proven to have improved electrochemical performance and stability under CO_2_ contained atmospheres. The author compared carbonate formation energies and implied that La_2_(CO_3_)_3_ and La_2_O_2_CO_3_ are less stable than SrCO_3_. Thus, La_2_NiO_4_ provides better CO_2_ resistance than SrCo_0.8_Nb_0.1_Ta_0.1_O_3‐δ_.^[^ ^9b^ ^]^

**Figure 15:**
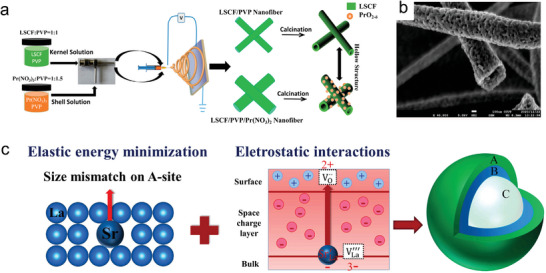
a) Flowchart for preparing LSCF nanofibers and LSCF@PrO_2‐δ_ core‐shell nanofibers. b) FE‐SEM images of LSCF@PrO_2‐δ_. Reprinted with permission.^[^ ^9c^ ^]^ Copyright 2022, American Chemical Society. c) Illustrations of driving dopant segregation to the surface, including elastic energy minimization, electrostatic interactions, and the formation of a core/bi‐shell structure. Reprinted with permission.^[^ ^146^ ^]^ Copyright 2023, Elsevier.

Some advanced fabrication methods, such as electrospinning, also utilized to fabricate core‐shell structured nano cathodes. Bai et al. concurrently electrospined LSCF and Pr(NO_3_)_3_ and co‐sintering at elevated temperatures to form LSCF@PrO_2‐δ_ (**Figure** **15**a,b), compared with the LSCF nanofibers, the LSCF@PrO_2‐δ_ core‐shelled nanofibers show improved surface areas due to the loose and porous morphology. These 3D core‐shell nanofibers effectively suppress Sr segregation on the surface and enhance air‐electrode stability, they also provide continuous pathways for charge transfer and extend the three‐phase boundaries, thereby improving the ORR kinetics.^[^ ^9c^ ^]^ Coating ≈5 nm ZrO_2_ onto the active air‐electrode La_0.6_Sr_0.4_CoO_3‐δ_ using atomic layer deposition (ALD) demsontrated remarkable stability of operating for 4000 h at 700 °C and the polarization resistance increases is much smaller than the pristine one. Besides, the chemical exchange between Co and Zr at the interface creates a negatively charged surface layer on the ZrO_2_ side and a positively charged layer on the cathode side. This charge separation at the junction repels the negatively charged $Sr_{La}^{\prime }$ ions and effectively suppressing Sr segregation.^[^ ^145a^ ^]^

Strategically segregate cations from material is feasible to in situ constructing core‐shell nanocatalysts. For intstance, doping aluminum into BaCo_0.4_Fe_0.4_Zr_0.1_Y_0.1_O_3‐δ_ B‐site results segregtion in several nanometer depth regions and forming a core/shell structure, the plenty of B‐site defects in the outmost shell plays a pivital role in enhaning charge transportion and electrode reaction.^[^ ^144a^ ^]^ Hou et al. developed a highly active core/bi‐shell nanolayer on La_1.2_Sr_0.8_Ni_0.5_Mn_0.5_O_4+δ_ material by utilizing A‐site cation segregation at high temperatures. They proposed that the key driving forces behind this segregation is the elastic and electrostatic interactions between the dopants and the surrounding lattice within the material (Figure 15c). This process leads to the forming of the core/bi‐shell, accompanied by the formations of B‐site deficient K_2_NiF_4_ phase and perovskite separately existing in two shells. The unique surface environment benefits electrocatalysts, oxygen/proton transfer and exchange, thus advancing the electrode reactions.^[^ ^146^ ^]^

### 5.4 Developing High/Medium Entropy Oxides

**Figure 16:**
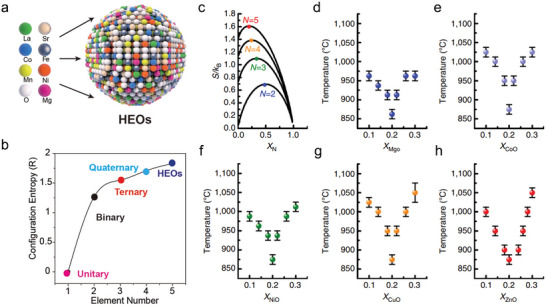
a) Schematic illustration of high‐entropy oxides. b) Configurational entropy as a function of the number of cations in the B‐site in (La_0.6_Sr_0.4_)(Co_0.2_M_0.8_)O_3_ material system, M represents Fe, Mn, Ni, Mg. Reprinted with permission.^[^ ^148l^ ^]^ Copyright 2022, John Wiley and Sons. c) The configurational entropy in solid solutions calculated as a function of mol% of the N^th^ component, and d–h) Phase diagrams showing the transition temperature to a single phase as a function of compositions, where maximum configurational entropy and lowest single phase formation temperatures are at near the equimolar point. Reprinted with permission.^[^ ^151^ ^]^ Copyright 2015, Springer Nature.

High‐entropy or medium‐entropy oxides are designed and demonstrated either with high electrochemical performance or improved stability under CO_2_ atmospheres (**Figure** **16**a,b).^[^ ^148^ ^]^ The formation of single or multi‐phase depends on the valence electron concentration, atomic size difference, electronegativity difference, and mixing entropy and enthalpy, where the former three aspects are materials’ intrinsic properties while the later mixing entropy and mixing enthalpy can be altered by external approaches.^[^ ^149^ ^]^ As mentioned above, the phase stability of a multi component system can be evaluated from the equation of ΔG_mix_ = ΔH_mix_‐TΔS_mix_. The configuration entropy (S_config_) for oxide can be calculated by the equation: ΔS_mix_ = ‐R∑*x_i_* ln*x_i_*, where R is the gas constant and *x_i_* is the mole fraction of *i*_th_ component.^[^ ^149a^ ^]^ It can be deduced that the equal molar fractions of each component would maximum mixing entropy.^[^ ^149^, ^150^ ^]^ Rost et al. verified the mixture stability by XRD measurements, the entropy can stabilize MgO, CoO, NiO, and ZnO mixing oxide at sufficient high temperatures with random cation occupancy, the deviation from equal molar fraction lead to the higher single phase transition temperatures as shown in Figure 16c–h, indicating a less stable phase comparing to that with an equal compositions.^[^ ^151^ ^]^ In terms of ABO_3_ perovskites, the configuration entropy can be written as S_config_ = ‐R $\left[\left(\sum_{a=1}^{n}x_{a}lnx_{a}\right)+\left(\sum_{b=1}^{n}x_{b}lnx_{b}\right)+3\left(\sum_{c=1}^{n}x_{c}lnx_{c}\right)\right]$, where *x_a_*, *x_b_*, and *x_c_* are the mole number of the ions at A, B and O sites, n is the number of components in the same position and R is ideal gas constant. Materials can be classified as high entropy (S_config_>1.5R), medium entropy (1R<S_config_<1.5R), and low entropy (S_config_<1R) based on the configuration entropy values.^[^ ^151^ ^]^

Xu et al. developed spinel oxide Fe_0.6_Mn_0.6_Co_0.6_Ni_0.6_Cr_0.6_O_4_ and used it for PCFC air‐electrode, the DFT calculation results indicate such high‐entropy oxide has closer O‐2p band center to Femi energy level and more negative O_2_ adsorption energy comparing to their individual components. In addition, such oxide also demonstrates good stability against CO_2_ at high temperatures, no phase change was observed after treating such sample at 600 °C for 12 h in 10 vol.% CO_2_ contained atmospheres, which concentration is 300 times higher than that in the air.^[^ ^148b^ ^]^ Liu et al. developed high‐entropy oxides consisting of six equimolar metals in the A‐site and formula as Pr_1/6_La_1/6_Nd_1/6_Ba_1/6_Sr_1/6_Ca_1/6_CoO_3‐δ_ (PLN‐BSCC) and demonstrated a high peak power density of 1.21 W cm^−2^ at 600 °C.^[^ ^148e^ ^]^ High‐entropy oxides rarely derive their properties solely from cation mixing entropy. Instead, the cation mixture typically involves factors such as size mismatches, differences in acidity, redox activity, and variations in the oxidation states of mixed cations. These factors collectively play critical roles in determining the stability and performance of the oxide. The B‐site high entropy oxide SrCo_0.5_Fe_0.2_Ti_0.1_Ta_0.1_Nb_0.1_O_3‐δ_ was developed and proved with enhanced stability under CO_2_ atmospheres due to their significantly depressed surface Sr segregation that resulted from the more negative value of ABE values. The different cation size inside the B‐site can result in lattice distortion and stress change around Sr cation, contributing to the sluggish Sr migration and suppress the Sr enrichment during operations.^[^ ^148a^ ^]^ Similar results can be found in medium entropy cathode Sr(Fe_α_Ti_β_Co_γ_Mn_ζ_)O_3‐δ_, high entropy cathode La_0.2_Pr_0.2_Nd_0.2_Sm_0.2_Sr_0.2_MnO_3‐δ_, and A‐site entropy engineered Pr_0.2_Ba_0.2_Sr_0.2_La_0.2_Ca_0.2_CoO_3‐δ_.^[^ ^148d,g,k^ ^]^

### 5.5 Self‐Assembly

**Figure 17:**
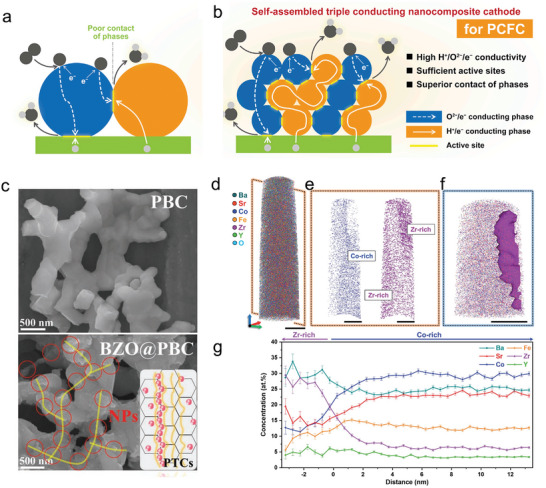
a) Conventional electron/ oxygen ion/ proton mixed‐conducting air‐electrode. b) self‐assembled electron/ oxygen ion/ proton mixed‐conducting nanocomposite air‐electrode. Reprinted with permission.^[^ ^5d^ ^]^ Copyright 2019, Elsevier. c) SEM pictures of PBC (top) and BZO@PBC (bottom) powders. Reprinted with permission.^[^ ^10b^ ^]^ Copyright 2022, John Wiley and Sons. d) 3D atomic map of the BSCFZY sample. e) Co‐BSCFZY (left hand) and Zr‐BSCFZY (right hand) phases illustrated by the Co and Zr atoms, respectively. f) Enlarged view of the top region with the iso‐concentration surface of 15 at% Zr. g) Proximity histogram obtained for the iso‐concentration surface. Reprinted with permission.^[^ ^10c^ ^]^ Copyright 2022, Royal Society of Chemistry.

The self‐assembly approach involves the design of materials that spontaneous form two or more phases during synthesis or post‐annealing processes, and have been widely applied on R‐PCCs (**Table** **3** and **Figure** **17**a,b). Typically, the main phase is responsible for providing charge‐carrier diffusion channels, such as the conduction of oxygen ions and holes,^[^ ^10^, ^152^ ^]^ or the concurrent conduction of oxygen ions, holes, and protons.^[^ ^153^ ^]^ Meanwhile, the minor phases surrounding the main phase play a crucial role in enhancing activity for oxygen dissociation and charge‐transfer processes. It was found developing nanocomposite air‐electrode Sr_0.9_Ce_0.1_Fe_0.8_Ni_0.2_O_3‐δ_ composed with 77.2 wt.% single perovskite main phase, a Ruddlesden‐ Popper phase (13.3 wt.%), a surface‐decorated NiO (5.8 wt.%), and CeO_2_ minor phase (3.7 wt.%). The unique composition enhances the electrochemical performance, attributing to the facilitated the oxygen bulk diffusion in Ruddlesden‐Popper phase, and promoted O^2−^ migration from surface to the bulk in the minor phases of NiO and CeO_2_.^[^ ^21a^ ^]^ Some nano‐catalysts exhibit enhanced catalytic performance but with low charge carrier conduction and are designed to function as minor phases. For instance, BaCoO_3‐δ_ is known to enhance ORR and OER performance at high temperatures, Ba_2_Co_1.5_Mo_0.25_Nb_0.25_O_6‐δ_ can decompose into BaCoO_3‐δ_ and a double perovskite Ba_2‐x_Co_1.5‐x_Mo_0.5_Nb_0.5_O_6‐δ_. A R‐PCC with this air‐electrode demonstrated remarkable electrochemical performance, achieving 1.17 W cm⁻^2^ and 2.04 A cm⁻^2^ at 1.3 V electrolysis voltage at 650 °C. DFT calculations confirm that the improved electrochemical performance is attributed to the facilitated oxygen surface catalysis by BaCoO_3‐δ_.^[^ ^10a^ ^]^

**Table 3:** Recent advances in R‐PCCs with self‐assembly method for enhancing air‐electrode performance at 600 °C.

Cell configuration [fuel‐electrode|electrolyte|air‐electrode]	Self‐assembled air‐electrode composition [major phase & minor phase(s)]	Minor phase size	Polarization resistance at open circuit voltages [Ωcm^2^]	Peak power density [mWcm^−2^]	Current density at 1.3 V [mA cm^−2^] and air‐electrode side reaction gas	Duration	Refs.
Ni‐BZCY172|BZCY172|PrBaFe_1.7_Mo_0.1_Ni_0.2_O_6‐δ_	PrBaFe_1.7_Mo_0.1_Ni_x_O_6‐δ_& NiO	≈100 nm	0.45	795	–	200 h @700 ^o^C	[153a]
Ni‐BZCYYb3511|BZCYYb3511|Ba_1.1_Gd_0.9_Co_2_O_6‐δ_	BaGdCo_2_O_6‐δ_& BaCoO_3_	–	0.4	604	–	600h@600 ^o^C	[153b]
Ni‐BZCYYb1711|BZCYYb1711|PrBaCo_1.92_Zr_0.08_O_5+δ_	PrBa_0.92_Co_1.92_O_5+ δ_& BaZrO_3_	∼200 nm	0.3	551	–	140h@600 ^o^C	[10b]
Ni‐BZCYYb1711|BZCYYb1711|BaCo_0.7_(Ce_0.8_Y_0.2_)_0.3_O_3‐δ_	BaCe_x_Y_y_Co_z_O_3‐δ_&BaCo_x_Ce_y_Y_z_O_3‐δ_ &BaCoO_3‐δ_	∼100 nm	0.11	743	–	800h@550 ^o^C	[5d]
Ni‐BZCYYb1711|BZCYYb1711|Ba(Co_0.7_Fe_0.3_)_0.7_(Ce_0.8_Y_0.2_)_0.3_O_3‐ δ_	Cubic phase BCFCY& rhombohedral phase BCFCY	≈300 nm	0.63	356	–	90h@550 ^o^C	[152]
Ni‐BZCYYb1711|BZCYYb1711|BaFe_0.6_Ce_0.2_Sc_0.2_O_3‐ δ_	Ce‐riched phase& Fe‐riched phase	≈100 nm	0.18	610	1180 (10%H_2_O/air)	110h@600 ^o^C	[154]
Ni‐BZCYYb1711|BZCYYb1711|Sr_0.9_Ce_0.1_Fe_0.8_Ni_0.2_O_3‐ δ_	Perovskite Sr_a_Ce_b_Fe_c_Ni_d_O_3‐ δ_& Ruddlesden‐Popper Sr_4_Fe_3_O_10_‐_δ_ & CeO_2_& NiO	–	0.2	531	364 (3%H_2_O/air)	120h@550 ^o^C	[41c]
Ni‐BZCYYb1711|BZCYYb1711|Ba_2_Co_1.5_Mo_0.25_Nb_0.25_O_6‐ δ_	Ba_2‐x_Co_1.5‐x_Mo_0.5_Nb_0.5_O_6‐ δ_& BaCoO_3_	∼100 nm	0.21	840	1340 (3%H_2_O/air)	1100h@550 ^o^C	[10a]
Ni‐BZCYYb1711|BZCYYb1711|Ba(Co_0.7_Fe_0.2_Zr_0.1_)_0.95_O_3‐ δ_‐Pr_6_O_11_	BaCo_0.7_Fe_0.2_Zr_0.1_ O_3‐ δ_& BaPrO_3_	–	0.5	340	–	100h@650 ^o^C	[155]
Ni‐BZCYYb1711|BZCYYb1711|PrNi_0.5_Mn_0.5_O_3‐δ_	PrNi_0.5_Mn_0.5_O_3‐δ_& PrO_x_	30 nm	0.3	440 (650 °C)	–	500h@700 ^o^C	[21c]
Ni‐BZCYYb1711|BZCYYb1711|PrBaCo_1.8_Nb_0.1_Y_0.1_O5+δ	PrBa_1‐x_Co_1.8_Nb_0.1‐x_Y_0.1‐x_O_5+δ_ & Ba_2_YNbO_6_	∼100 nm	0.19	970	2200 (3%H_2_O/air)	120h@600 ^o^C	[156]
Ni‐BZCYYb1711|BZCYYb1711| Ba_1.5_Sr_1.5_Co_1.6_Fe_0.4_O_7‐δ_	Cubic Ba_0.5_Sr_0.5_Co_0.8_Fe_0.2_O_3‐δ_ & hexagonal phase Ba_4_Sr_4_(Co_0.8_Fe_0.2_)_4_O_16‐δ_	–	0.26	1670	2720 (10%H_2_O/air)	200h@600 ^o^C	[157]
Ni‐BZCYYb1711|BZCYYb1711| Ce_0.2_Ba_0.2_Sr_0.2_La_0.2_Ca_0.2_CoO_3‐δ_	Ce_0.2‐y_Ba_0.2_Sr_0.2‐x_La_0.2‐x_Ca_0.2_CoO_3‐δ_& CeO_2_& La_0.5_Sr_0.5_CoO_3‐δ_	∼∼200 nm	0.23	1660	1760 (3%H_2_O/air)	200h@600 ^o^C	[158]

BZCY172: BaZr_0.1_Ce_0.7_Y_0.2_O_3‐δ_; BZCYYb3511: BaZr_0.3_Ce_0.5_Y_0.1_Yb_0.1_O_3‐δ_; BZCYYb1711: BaZr_0.1_Ce_0.7_Y_0.1_Yb_0.1_O_3‐δ_.

In addition to enhancing surface activity, minor phases also provide essential charge‐carrier transport paths, broadening the reaction zone and boosting electrochemical performance, or performing other specific functions. It has been found that Ruddlesden‐Popper structured oxides exhibit the ability to transport proton.^[^ ^41^, ^138^ ^]^ Inspired by this, Song et al. in situ formed an RP‐structured phase enriched with CeO₂ and NiO nanoparticles. The resulting PCFCs with this electrode achieved a high output performance of 745 mW cm⁻^2^ at 650 °C, attributed to enhanced hydration and proton transfer in the RP phase.^[^ ^41c^ ^]^ Similarly, high‐temperature calcination of BaCo_0.7_(Ce_0.8_Y_0.2_)_0.3_O_3‐δ_ resulted in the formation of a nanocomposite consisting of H⁺/e⁻‐conducting BaCe_x_Y_y_Co_z_O_3‐δ_ and mixed O^2^⁻/e⁻‐conducting BaCo_x_Ce_y_Y_z_O_3‐δ_. The proton and oxygen ion conductivities, as well as transference numbers were measured using the hydrogen/oxygen permeation method. The results showed that proton conductivity and transference numbers were higher than those for oxygen ions, suggesting that BaCo_0.7_(Ce_0.8_Y_0.2_)_0.3_O_3‐δ_ is a highly promising cathode material for PCFCs. This composite electrode also exhibited increased electronic conductivity, reduced TEC, and an increased number of reaction sites.^[^ ^5d^ ^]^ In a similar approach, incorporating ZrO_2_ into the double perovskite PrBaCo_2_O_5+δ_ (PBC) successfully in situ constructed PrBaCo_2_O_5+δ_ coated by proton conducting BaZrO_3_ (BZO) nanocomposites through the liberation of A‐site cations. The application of this electrode improved the electrochemical performance of PCFC from 781 mW cm^−2^ that with PBC cathode to 1453 mW cm^−2^ at 700 °C (Figure 17c).^[^ ^10b^ ^]^ Thermal expansion is one of the crucial properties of air‐electrodes. A large difference in thermal expansion coefficient with the electrolyte will cause electrode‐electrolyte delamination and affect stable operation. In a case study of Ba_0.5_Sr_0.5_Co_0.6_Fe_0.2_Zr_0.1_Y_0.1_O_3‐δ_ (BSCFZY) air‐electrode, it spontaneously forms two distinct cubic perovskites, a Co‐rich Ba_0.5_Sr_0.5_Co_0.7_Fe_0.2_Zr_0.07_Y_0.03_O_3‐δ_ phase, which enhances electrocatalytic activity, and a Zr‐rich Ba_0.6_Sr_0.4_Co_0.3_Fe_0.2_Zr_0.4_Y_0.1_O_3‐δ_ phase that supports microstructural robustness. The thermal expansion coefficient (TEC), calculated from XRD results, was found to be 1.9×10⁻^5^ K⁻^1^ for Co‐BSCFZY and 1.36×10⁻^5^ K⁻^1^ for Zr‐BSCFZY. The lower TEC value for Zr‐BSCFZY likely results from the fixed valence state of Zr ions, contributing to stable operations. Notably, XPS results showed that non‐lattice Sr is significantly lower than lattice Sr, indicating minimal Sr segregation on the surface of BSCFZY (Figure 17d–g).^[^ ^10c^ ^]^

### 5.6 in situ Exsolve Active Nanoparticles

The B‐site cations often transition metal elements that can be reduced in reduction atmospheres, can exsolve from the lattice when the cation diffusion energy is satisfied. These nanoparticles serve as the active sites for surface catalytic reactions, thereby enhancing the electrochemical performance (**Table** **4**). In previous researches, the in situ formation of nanoparticles has frequently been employed in fuel electrodes and applied in hydrocarbon fueled fuel cells,^[^ ^159^ ^]^ and steam/CO_2_ electrolysis,^[^ ^160^ ^]^ where a reduction atmosphere predominates to stabilize the exsolved nanoparticles. Similar to the segregation of inert cations to the surface, the exsolution process of B‐site cations also influenced by lattice defects (e.g., A‐site deficiency and lattice strain) and external environments (e.g., temperatures and oxygen partial pressures). However, the situation becomes more complex for air‐electrodes operating in oxidative atmospheres, as the newly formed nanoparticles can reincorporate into the host oxide after annealing at high temperatures in an oxidizing atmosphere or transform into metal oxides through oxidation.^[^ ^161^ ^]^

**Table 4:** Recent advances in R‐PCCs with in situ exsolve nanocatalysts for enhancing air‐electrode performance at 600 °C.

Cell configuration [fuel electrode|electrolyte|air‐electrode]	Exsolved nanocatalyst and size	Exsolution method	Polarization resistance at open circuit voltages [Ωcm^2^]	Peak power density [mWcm^−2^]	Current density at 1.3 V [mA cm^−2^] and air‐electrode side reaction gas	Duration	Refs.
Ni‐BZCYYb1711|BZCYYb1711|Ba_0.95_(Co_0.4_Fe_0.4_Zr_0.1_Y_0.1_)_0.95_Ni_0.05_O_3‐δ_	NiO, ≈100 nm	Point defects induced	≈0.1	750	–	400h@550 ^o^C	[11d]
Ni‐BZCYYb1711|BZCYYb1711|Pr_2.7_Ni_1.6_Cu_0.3_Nb_0.1_O_7‐ δ_	NiO, ≈30 nm	Point defects induced	0.25	1024	–	130h@600 ^o^C	[169]
Ni‐BZCYYb1711|BZCYYb1711| Ba(Co_0.4_Fe_0.4_Zr_0.1_Y_0.1_)_0.95_Ni_0.05_F_0.1_O_2.9‐δ_	NiO, ≈500 nm	Point defects induced	0.17	779	1089 (3% H_2_O/air)	100@550 ^o^C	[170]
Ni‐BZCYYb1711|BZCYYb1711|PrSrCo_1.8_Nb_0.2_O_6‐ δ_	SrCo_0.5_Nb_0.5_O_3‐ δ_, ∼150 nm	Temperature induced	0.3	940	∼1500 (10%H_2_O/air)	120h@600 ^o^C	[21b]
Ni‐BZCYYb1711|BZCYYb1711| Ba_0.8_Gd_0.8_Pr_0.4_Co_2_O_5+δ_	Gd_x_Co_y_O_3‐δ_, N/A	Temperature induced	0.27	909	2336 (3% H_2_O/air)	100h@ 600 ^o^C	[171]
Ni‐BZCYYb1711|BZCYYb1711|Ba_0.95_Ag_0.05_Co_0.4_Fe_0.4_Zr_0.1_Y_0.1_O_3‐δ_	Ag, ≈15 nm	Water induced	0.11	778	–	603h@500 ^o^C	[19b]
Ni‐BZCYYb1711|BZCYYb1711|Ba_0.9_Pr_0.1_Co_0.7_Fe_0.2_Y_0.1_O_3‐δ_	BaCoO_3_, N/A	Water induced	0.43	500	–	85h@700 ^o^C	[172]
Ni‐BZCYYb1711|BZCYYb1711|PrBa_0.8_Ca_0.2_Co_2_O_5+δ_	BaCoO_3_, N/A	Water induced	0.24	1060	1510 (3% H_2_O/air)	500h@650 ^o^C	[15b]
Ni‐BZCYYb1711|BZCYYb1711|PrBaCo_1.6_Fe_0.2_Nb_0.2_O_5+ δ_	Nb‐deficient PrBaCo_1.6_Fe_0.2_Nb_0.2‐x_O_5+ δ_, N/A	Water induced	0.8	723	1000 (3% H_2_O/air)	100h@ 650 ^o^C	[15c]
Ni‐BZCYYb1711|BZCYYb1711| (Nd_0.5_Ba_0.5_)_0.95_Mn_0.7_Co_0.15_Ni_0.15_O_3‐δ_	(Co_x_Ni_y_)_3_O_4_, 10 nm	Chemical reduction	0.38	730	1110 (3% H_2_O/air)	100h@ 600 ^o^C	[173]

BZCYYb1711: BaZr_0.1_Ce_0.7_Y_0.1_Yb_0.1_O_3‐δ_.

Point defects can induce B‐site cation exsolution. Design perovskites with non‐stoichiometry usually demonstrated with enhanced electrochemical performance, which mainly results from the loosely packed cations in the bulk material and the unexpected surface defects result from the cation mismatch. The creating of A‐site deficiency to the enhanced electrochemical performance has already been proved in many reaction systems.^[^ ^11a–d^ ^]^ It was found that A‐site deficient La_0.6_Sr_0.4_Co_0.2_Fe_0.8_O_3‐δ_ with Sr deficiency lead to the improved ionic conductivity and catalytic property for ORR.^[^ ^162^ ^]^ Similar results were found in (Ba_0.5_Sr_0.5_)_1‐x_Co_0.8_Fe_0.2_O_3‐δ_ and PrBaCo_2_O_5+δ_.^[^ ^13^, ^163^ ^]^ Liang et al. achieved exsolving nickel oxide by creating A‐site deficiency in the perovskite material Ba_0.95_(Co_0.4_Fe_0.4_Zr_0.1_Y_0.1_)_0.95_Ni_0.05_O_3‐δ_. 5% atomic A‐site cation deficiency induces NiO segregation to the surface and shows stability under pure O_2_ at 550 °C for 100 h. The exsolved NiO layer was proved to have positive effects to ORR reactions, and the single cell assembled with this cathode demonstrated a peak power density of 540 mW cm^−2^ at 550 °C and operates smoothly for 400 h.^[^ ^11d^ ^]^

**Figure 18:**
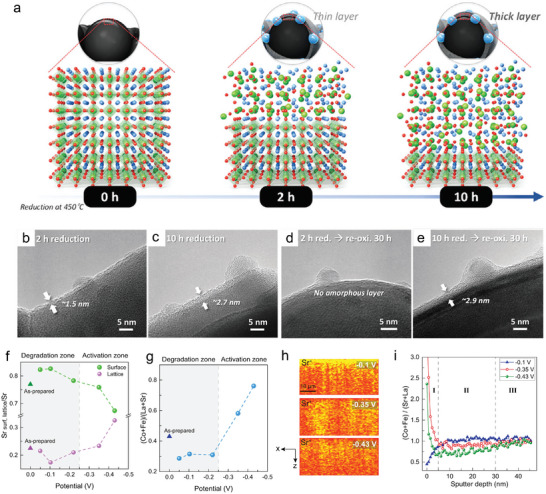
a) Schematic illustration of change of crystallinity according to the reduction time. TEM image of thickness and stability of amorphous layer in LSCF‐Cu according to the reduction time. b) reduced for 2 h, c) 10 h, d) after reoxidation of 2 h reduced sample, and e) after reoxidation of 10 h reduced sample. The reduction and reoxidation was conducted in a condition of 4% H_2_ at 450 °C, and in air at 650 °C for 30 h, respectively. Reprinted with permission.^[^ ^165^ ^]^ Copyright 2024, John Wiley and Sons. f) Sr concentrations on surface and lattice that normalized to total Sr as a function of potential in LSCF‐SDC. g) The surface (Co+Fe)/(La+Sr) cations ratios under various potentials. h) Reconstructed cross‐section images of Sr intensity under various potentials. i, Depth‐profile of (Co+Fe)/(La+Sr) ratio for LSCF‐SDC electrodes performed under various potentials. Reprinted with permission.^[^ ^85^ ^]^ Copyright 2023, Springer Nature.

The reduction atmosphere also induced nanoparticle exsolutions on cathode materials. Reported results indicate silver can be exsolved by reducing Sr_0.95_Ag_0.05_Nb_0.1_Co_0.9_O_3‐δ_ material in 10% H_2_ at 320 °C for 1 h. The exsolution of silver enhanced cathode electrochemical performance by decreasing polarization resistance and activation energy. The fuel cells assembled with this air‐electrode shows superior peak power density of 1116 mW cm^−2^ at 500 °C. Detailed analysis on polarization resistance indicates the low‐frequency resistance decreased greatly, which is related to oxygen surface process, indicating the exsolution of silver nanoparticles accelerated oxygen adsorption‐desorption, oxygen dissociation, or the surface diffusion of intermediate oxygen species.^[^ ^11e^ ^]^ Similar results were found in Ba_0.95_Ag_0.05_Co_0.9_Ta_0.1_O_3‐δ_ material, the 4% H_2_/Ar at 300 °C for 2 h treatment which referred by H_2_‐TPR results, triggers the silver exsolutions and boosts the catalytic performance and stability.^[^ ^11f^ ^]^ The thermodynamic stability of exsolved nanoparticles can be referred to Ellingham diagram, where Ag is among the few elements that can be stabilized under oxidative atmospheres and with improved catalytic performance toward ORRs, while in terms of Co, Fe, and Ni, they only have their oxides in oxidative atmosphere at high temperatures, and these elements may also encounter phase change with the change of *p*O_2_ and temperature, further increasing the complexity of surface property analysis.^[^ ^164^ ^]^ Jeon et al. conducted studies on the chemical reduction of La_0.6_Sr_0.4_(Co_0.2_Fe_0.8_)_0.97_Cu_0.03_O_3‐δ_ (LSCF‐Cu) for high‐temperature ORR. They found that an amorphous layer forms at low temperatures between 100—200 °C, while nanoparticles are exsolved at ≈400 °C (**Figure** **18**a). When these materials operate in oxidation atmospheres, the electrode polarization resistance increased by 27% over 90 h operation on unreduced LSCF‐Cu, which has similar degradation rates with the LSCF‐Cu reduced at 200 °C for 2 h and 450 °C for 2 h. This is because amorphous layer vanished upon re‐crystallization in the oxidation atmosphere, rendering them unable to suppress Sr segregation. In contrast, LSCF‐Cu reduced at 450 °C for a longer time of 10 h demonstrated stable performance for 100 h, attributing to the persistence of the amorphous layer (Figure 18b–e).^[^ ^165^ ^]^

Electrochemical bias can alter electrode *p*O_2_ and charge carrier concentration that near electrode/electrolyte interface, consequently, such electrochemical bias would induce cation exsolution and form multiphase catalysts.^[^ ^166^ ^]^ Compared to defect‐induced or atmosphere‐induced exsolution methods, electrochemical bias‐induced exsolution offers advantages such as faster exsolution speeds and lower energy consumption. The main driving force for exsolution is the *p*O_2_ gradient between the oxide lattice and the external environment. However, reduction gas diffusion into the oxide requires time, which depends on factors like material morphology, diffusion coefficients, and temperature. In contrast, electrochemical reduction using external voltages can achieve a very low *p*O_2_ with minimal applied voltage, enabling a much faster exsolution process at relatively lower temperatures.^[^ ^167^ ^]^ For instance, to achieve the same level of oxygen loss, chemical reduction using H_2_ requires over 15 h, whereas electrochemical reduction achieves the same result in just 150 s.^[^ ^167a^ ^]^ Additionally, electrochemical reduction allows fine tailoring of the catalyst surface and facilitates deeper reduction depths. Experimental results show that electrochemical reduction on La_0.43_Ca_0.37_Ni_0.06_Ti_0.94_O_3‐δ_ produces significantly higher particle populations, smaller particle sizes, and greater reduction depths.^[^ ^167a^ ^]^ Although electrochemical reduction‐triggered exsolution of active nanoparticles were first investigated on fuel electrodes, it holds particular significance for being application on air‐electrodes. This is because air‐electrodes, which contain abundant oxygen vacancies and weak cation‐oxygen bonds, are prone to lattice collapse when subjected to abrupt reductions in reducing atmospheres. To prevent lattice collapse, chemical reduction is typically conducted at lower temperatures of ≈400 °C and hold for a long time for air‐electrodes.^[^ ^11e,f^ ^]^ The electrochemical bias induced cation segregation was applied on double perovskite PrBaCo_2_O_5+δ_, research results indicate the anodic polarization triggers BaO formation on the surface and leads to the electrochemical performance degradation, while the cathodic polarization depletes such BaO then contributing to the electrochemical performance reactivation.^[^ ^168^ ^]^ A real case of demonstrating such method to improve the stability in ORR and electrochemical activity on a porous electrode is made of La_1‐x_Sr_x_Co_1‐x_Fe_x_O_3_‐Sm_0.2_Ce_0.8_O_1.9_. The XPS results shown with applying negative bias, the surface Sr is depressed while the (Co+Fe)/(La+Sr) ratio is increased when the applied voltage is higher than (more negative) −0.25 V, forming an active Co/Fe surface (Figure 18f,g). *Depth‐profiling* ToF‐SIMS experiments reveals such active layer is ≈5 nm and higher intensity of Co/Fe on the surface can be obtained by applying higher electrochemical reductions (Figure 18h,i).^[^ ^85^ ^]^ An application of this method on fuel cells was demonstrated using Ln_0.2_Ba_0.8_Co_0.7_Fe_0.3_O_3‐δ_ (Ln = La, Pr, Nd) materials, the authors found that through applying ‐2 V bias on fuel cell can trigger CoO formation and significantly reduce polarization resistance.^[^ ^86^ ^]^

### 5.7 Predicting and Interpretation of Surface Properties using Machine Learning

Machine learning (ML) can be applied in two key areas: materials exploration and fundamental understanding of inorganic oxides.^[^ ^174^ ^]^ In the former and in R‐PCCs, ML has been utilized to identify efficient air‐electrodes^[^ ^175^ ^]^ and oxides with specific properties such as proton concentrations.^[^ ^174^, ^176^ ^]^ In the latter, ML is employed to deepen our understanding of surface structures and reaction mechanisms.^[^ ^177^ ^]^

As previous section mentioned, the proton uptake ability is of great importance for electrode surface evolutions and reconstructions. In the search for oxides with super proton concentrations, the number of possible ABO_3_ perovskites is vast. This makes the conventional trial‐and‐error approach for developing proton‐conducting oxides highly challenging due to the low success rate. Hyodo et al. applied ML techniques to identify AB_1‐x_B’_x_O_3‐δ_ oxides with superior proton uptake abilities. Their prediction was conducted for 8613 hypothetical compounds comprising 78 different hosts with various A, B, and B’ cations. For training data, they evaluated 22 perovskite compounds to reliably determine proton concentrations across different chemical compositions, temperatures, and water partial pressures. The study demonstrated that gradient boosting regressor (GBR) models exhibited the best predictive performance. The most effective descriptors included temperature, structural and chemical parameters such as perovskite type, host compound chemistry, dopant concentration, and structural symmetry. The model nominated SrSnO_3_ as a promising host. Experimentally, SrSn_0.8_Sc_0.2_O_3‐δ_ demonstrated both high proton incorporation and conduction. The proton conductivities of SrSn_0.8_Sc_0.2_O_3‐δ_ were found to be higher than those reported La based perovskites, Sc‐doped BaTiO_3_, and Y doped SrZrO_3_.^[^ ^176a^ ^]^ When applying ML to develop new oxides, a significant challenge lies in the shortage of consistent and sufficient experimental data. Additionally, when using data from DFT calculations, such computational explorations of materials are often based on ideal, defect‐free states, limiting their applicability. Fujii et al. addressed these issues by implementing a sequential high‐throughput computational approach combined with physically interpretable ML that incorporates defect chemistry across a wide range of oxides. The constructed ML models successfully quantified the impacts of structural and chemical features on dopant dissolution and hydration. Notably, their work identified the sillenite‐structured Pb‐doped Bi_12_SiO_20_ as a promising proton conductor, exhibiting unique and fast 3D proton conduction along a loosely bonded BiO_5_ network.^[^ ^176b^ ^]^

One challenge in predicting catalytic activity lies in identifying suitable descriptors. For example, catalytic activity has been shown to correlate with the d‐band center for metals and the oxygen 2p‐band center for oxides.^[^ ^14^, ^178^ ^]^ Additionally, factors such as e.g. orbital filling,^[^ ^179^ ^]^ spin state,^[^ ^180^ ^]^ and magnetic ordering have also been found to correlate with the catalytic activity of materials.^[^ ^180^, ^181^ ^]^ To reduce computational costs, Jacobs et al. developed ML models based on elemental features. They utilized a database containing 749 data points spanning 299 perovskite compositions. The perovskites in the database include 37 elements, the dataset also includes surface exchange coefficients, oxygen diffusion coefficients, and ASR values. They show these ML models are orders of magnitude faster to evaluate electrochemical performance than using the O 2p‐band center descriptor because DFT calculations for each material is not needed.^[^ ^182^ ^]^

**Figure 19:**
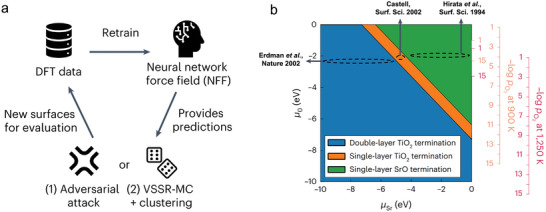
a) The flowchart for training NFF model, the process begins with existing DFT datasets. b) Computed phase diagrams under different O and Sr chemical potentials and external conditions. Reprinted with permission.^[^ ^12c^ ^]^ Copyright 2023, Springer Nature.

ML can be integrated with molecular dynamics (MD), Monte Carlo (MC), and DFT calculations to model large and complex systems at the molecular scale. This approach enhances the accuracy of these methods and provides deeper insights into material property changes. MD were used for calculate the surface phase digram with the combination of ML to improve accuracy at elevated temperatures.^[^ ^177a^ ^]^ Timmermann at al. utilized machine‐learned interatomic potentials, which can be trained with a limited number of DFT calculations, to identify the most stable surface terminations of IrO_2_ and RuO_2_. The process starts with creating a reference database of DFT structures to train a nonparametric ML Gaussian Approximation Potential (GAP). GAP models break down the system's total energy into a sum of atomic energies based on the local chemical environment. The GAP predictions indicated that the most stable structures are (101) and (111) (1×1) under simulated annealing conditions. These predictions were further validated through DFT calculations, as well as low‐energy electron diffraction (LEED) and STEM analysis on annealed IrO₂ catalysts.^[^ ^12a^ ^]^ While ML offers the advantage of significantly faster calculations than DFT with comparable accuracy, it faces difficulties in autonomously exploring the phase space of multicomponent surfaces. To address this, Du et al. developed an Automatic Surface Reconstruction (AutoSurfRecon) framework that combines active learning (AL) and ML‐based force fields for rapid and precise energetic calculations. They validated their approach by successfully recovering GaN (0001) and Si (111) with known reconstruction surfaces using classical force fields. Furthermore, they demonstrated the framework's effectiveness on the more complex perovskite SrTiO_3_ (001) surface, which is difficult to calculate with classical force fields. By utilizing a neural network force field (NFF) energy model and fewer than 5000 DFT single‐point calculations, the NFF was trained to accurately predict energies across various chemical compositions. This approach allowed to reconstruct a surface phase diagram for SrTiO_3_ (001) that aligned well with existing literature and revealed previously unknown low‐energy surface terminations (**Figure** **19**a,b).^[^ ^12c^ ^]^

In addition to thermodynamic predictions, ML is also employed to predict surface reconstruction kinetic pathways. Yoon et al. introduced CatGym, a deep reinforcement learning (DRL) environment, to identify thermal surface reconstruction pathways and their associated kinetic barriers in crystalline solids under reaction conditions. This approach enables the exploration of potential surface segregation phenomena and their corresponding transition states, addressing the challenge of predicting catalyst stability.The researchers trained their DRL agent on a ternary Ni_3_Pd_3_Au_2_ (111) alloy catalyst. In the CatGym environment, the agent not only explores a broader range of local and global minima configurations compared to traditional methods but also generates kinetic pathways to reach those configurations.^[^ ^12b^ ^]^ Meldgaard et al. combined image recognition and reinforcement learning to determine the atomic structure of reconstructed crystalline surfaces. They incorporated configurational information into an image‐like representation of the structure, allowing a convolutional neural network to sequentially place atoms and build the global minimum structure. Finally, they demosntrated the effectiveness of this method and solve the complex SnO_2_ (110) reconstruction.^[^ ^183^ ^]^

## 6 Conclusion and Perspectives

### 6.1 Conclusion

In R‐PCCs and similar electrochemical devices, electrode surface evolution typically governs catalytic properties and overall output performance. This review provides a comprehensive summary of air‐electrode surface evolution, encompassing the thermodynamics and kinetics of surface evolution, the underlying principles of surface degradation, key characterization techniques, and recent advancements in performance enhancement. Overall, air‐electrode surface reconstruction is primarily governed by thermodynamic principles when sufficient time is allowed, but it can be kinetically accelerated at elevated temperatures. Cation diffusion within the grain interior is several orders of magnitude slower than at the grain boundaries and exhibits lower activation energies. Although the cation diffusion coefficients are significantly lower than those of lattice oxygen or protons, preventing such diffusion generally requires temperatures below 500 °C.

We show that electrochemical characterizations, combined with reaction models and model electrodes, are the most used methods to decode surface reactions and evolutions. However, directly obtaining surface information requires techniques such as FTIR, Raman, XPS, microscopy, LEIS, and ToF‐SIMS. These techniques offer specific detection ranges, from outmost atomic layer to hundreds of nanometers, and can provide diverse information, from electron signals to cation distributions. Some of these methods can be operated under ambient pressure and allow for in situ characterizations using specially designed reaction chambers. However, very limited studies have successfully performed dual‐chamber characterizations on R‐PCCs due to challenges related to gas sealing.

Inactive cation segregation, poisoning by external gases, and cation interdiffusions between neighborhood components are main contributors to the electrode surface deterioration. These reactions are complex and generally exacerbated at high temperatures and presence of water. A key challenge in understanding these evolution mechanisms is establishing the relationship between materials properties and external conditions. In practical conditions, multiple factors may influence surface properties simultaneously. For example, the presence of water on an air‐electrode can lead to changes in lattice strain, alter point defects by filling oxygen vacancies, and modify electrostatics. Identifying the primary factor that drives surface evolution is crucial and requires a thorough understanding of surface evolution mechanisms and well‐designed experiments. The presence of water can enhance electrochemical performance on some air‐electrodes. Proton uptake at the air electrode forms surface protons and bulk proton diffusion channels, which are believed to expand the reaction area and provide additional active sites. Furthermore, the unique proton uptake ability has been shown to induce active cation surface segregation and form active nanocatalysts, further improving electrochemical performance.

Key strategies to stabilize and enhance surface activity include doping, developing oxides beyond perovskites, creating high‐entropy oxides, fabricating core‐shell nanocatalysts, self‐assembly, in situ exsolving nanoparticles, and leveraging AI to predict and understand surface properties. Using these methods typically face challenges of balancing stability and catalytic performance. For instance, doping less reducible cations could enhance electrode stability yet typically show less activity, covering the surface with stable oxides to form core‐shell structured cathodes may extend the lifetime of R‐PCCs but could also alter charge‐transfer barriers in step reactions, resulting in lower electrochemical performance. Similarly, in situ exsolve nanoparticles can significantly enhance surface activity, but excessive exsolution may cause lattice shrinkage and disrupt oxygen diffusion pathways. The key to achieving overall electrochemical performance enhancement in R‐PCCs is improving surface reaction kinetics without damaging other properties.

### 6.2 Future Perspectives

In the future design of R‐PCC air electrodes, more attentions should be focused on developing in situ characterization techniques, understanding crystal limits when strategically performing surface reconstructions, analyzing surface‐to‐bulk property evolutions, and advancing AI technologies. The advancement of key components has enabled certain characterization techniques to operate under ambient pressures and high temperatures. The in situ characterization systems integrate electrochemical workstations, heating units, gas supply units, single cells, and various characterization techniques. Previous in situ characterizations have primarily been conducted on thin films and model electrodes. The development of reaction chambers for real R‐PCCs that operate with two gas chambers is of great importance, because the effects of electrochemical potential and chemical potential on the electrode remain to be explored.

Surface evolution is closely linked to the bulk properties of the electrode, the lattice structure can collapse if excessive cations are extracted or segregated to the surface. Consequently, a key challenge in leveraging surface reconstruction to enhance electrochemical performance lies in understanding the crystal limits to strategically control exsolved cations without causing lattice collapse. It is difficult to precisely tailor electrode surface properties by altering reaction gases or temperatures due to complexity of mass and heat transfer inside the electrode, thus utilizing an external bias to trigger cation segregation becomes a promising approach for efficiently and precisely reconstructing the electrode surface. Another critical point is understand surface‐to‐bulk property evolutions. The term “electrode surface” is not always precisely defined, and in some cases, sub‐layers significantly influence the catalytic performance of the outermost layer. Surface evolution is often accompanied by changes in sub‐layer properties. For example, when inactive cations segregate to the surface, they can create a sub‐layer enriched with active cations. Understanding these surface‐to‐bulk evolutions, including cation distributions, defect types, and electronic structures, are particularly crucial in heterogeneous electrocatalysts. Techniques such as polishing or beam etching are needed to expose these sub‐layers and reveal the surface‐to‐bulk property changes. This understanding is crucial for deciphering surface evolution mechanisms and identifying true active sites for ORR and OER.

Advancing AI to improve material development efficiency and deepen our understanding of material properties is crucial in today's R‐PCC development. Much of our current understanding of mechanisms relies heavily on computational calculations. However, due to limitations in computational resources, simulated conditions are often idealized and differ significantly from real‐world scenarios. AI technologies, such as machine learning combined with molecular dynamics and density functional theory calculations, can significantly reduce computational costs while improving accuracy. The application of AI tools for predicting new materials will play an increasingly vital role due to the rising cost of labor and the rapid advancement of fuel cell technology. Besides algorithm development, generating reliable, consistent, and large‐scale experimental data for training these AI tools should be a key focus.

## Conflict of Interest

The authors declare no conflict of interest.
